# On the Consistency between Gene Expression and the Gene Regulatory Network of *Corynebacterium glutamicum*

**DOI:** 10.1089/nsm.2020.0014

**Published:** 2021-03-08

**Authors:** Doglas Parise, Mariana Teixeira Dornelles Parise, Evans Kataka, Rodrigo Bentes Kato, Markus List, Andreas Tauch, Vasco Ariston de Carvalho Azevedo, Jan Baumbach

**Affiliations:** ^1^ TUM School of Life Sciences, Technical University of Munich, Freising-Weihenstephan, Germany.; ^2^ Institute of Biological Sciences, Universidade Federal de Minas Gerais, Belo Horizonte, Brazil.; ^3^ Center for Biotechnology (CeBiTec), Bielefeld University, Bielefeld, Germany.

**Keywords:** *Corynebacterium glutamicum*, Gene Regulatory Networks, inconsistency assessment, systems biology

## Abstract

**Background:** Transcriptional regulation of gene expression is crucial for the adaptation and survival of bacteria. Regulatory interactions are commonly modeled as Gene Regulatory Networks (GRNs) derived from experiments such as RNA-seq, microarray and ChIP-seq. While the reconstruction of GRNs is fundamental to decipher cellular function, even GRNs of economically important bacteria such as *Corynebacterium glutamicum* are incomplete.

**Materials and Methods:** Here, we analyzed the predictive power of GRNs if used as in silico models for gene expression and investigated the consistency of the *C. glutamicum* GRN with gene expression data from the GEO database.

**Results:** We assessed the consistency of the *C. glutamicum* GRN using real, as well as simulated, expression data and showed that GRNs alone cannot explain the expression profiles well.

**Conclusion:** Our results suggest that more sophisticated mechanisms such as a combination of transcriptional, post-transcriptional regulation and signaling should be taken into consideration when analyzing and constructing GRNs.

## Introduction

Bacterial genomes are small and compact; on average, 88% of their genomes consist of coding genes.^1^ Consequently, the ability to regulate gene expression in diverse environments is crucial for stabilizing cell homeostasis and adapting to environmental challenges.^2^ Computational systems biology uses Gene Regulatory Networks (GRNs) to understand the mechanisms that coordinate the shifts in gene expression and to represent the transcriptional gene regulation of organisms. These networks are consistently expanding our understanding of how the genotype manifests in the phenotype of an organism. Computationally, GRNs are modeled as directed graphs with nodes representing genes and edges or links representing the interactions between regulators, also known as transcription factors (TFs), and their target genes (TGs).^3,4^

Techniques to measure gene expression levels and infer GRNs include microarrays,^5^ ChIP-seq,^6^ and RNA-Seq.^7^ Microarrays measure the expression levels of known genes through the quantification of the fluorescence emitted by chemically marked complementary DNA attached to a solid surface. Microarrays can also determine the binding site of TFs when combined with chromatin immunoprecipitation.^8,9^ ChIP-seq is also used to determine the TF binding sites by sequencing DNA fragments that are bound to the TFs during chromatin immunoprecipitation, and is then used to map them to a reference genome.^6^ RNA-Seq is used to quantify the entire set of RNA in a biological sample at a particular moment through high-throughput sequencing.^7^ Several methods to infer GRNs from such gene expression data have been developed and evaluated^10–12^; these networks are commonly modeled as Boolean and Bayesian networks.^13–16^ These methods have been applied to reconstruct experimental GRNs and have resulted in multiple databases and online platforms for the analysis of model organisms. Such resources include RegulonDB,^17^ Subtiwiki,^18^ CoryneRegNet,^19^ and Abasy Atlas,^20^ for *Escherichia coli*, *Bacillus subtilis*, *Corynebacterium glutamicum*, and several organisms, respectively. Previous evaluations of gene expression-based methods to infer GRNs demonstrated a moderate performance on experimental microarray data and a better performance on *in silico*-generated gene expression data.^10,11^ Inference methods developed for both bulk and single-cell data were evaluated with single-cell transcriptomic data, and reached the conclusion that the algorithms performed poorly using both experimental or *in silico*-generated data.^21,22^

Despite the importance of GRNs and the low performance of GRN inference methods, few studies systematically evaluated the consistency of these networks with gene expression data. In 2003, Gutiérrez-Ríos et al.^23^ assessed the consistency of the *E. coli* GRN in a few well-studied genes. Later, Siegel et al.^24^ developed a mathematical framework evaluating the consistency in interaction graphs; they used an *in silico* experiment derived from experimental literature data of gene and metabolic networks to test their approach. Based on this framework, Guziolowski et al.^25^ analyzed the consistency of the *E. coli* GRN using three independent microarray datasets and ascertained the inconsistency of the network. Guziolowski et al. developed BioQuali,^26^ a Cytoscape app for detecting inconsistencies in GRNs and suggesting changes that would restore the network consistency with the user-provided expression data. Other Cytoscape apps such as contradictions in microarrays,^27^ CytoASP,^28^ and SigNetTrainer^29^ assess the consistency of interaction networks and expression data from a single study. The first makes use of Boolean network models to detect inconsistencies in interaction networks. CytoASP^28^ uses logical roles through Answering Set Programming to identify inconsistencies and to suggest how to repair them. SigNetTrainer^29^ uses Integer Linear Programming to detect and remove inconsistencies from the networks. Collectively, these pioneer works allowed researchers to evaluate the consistency of the existing GRNs and their gene expression data. Furthermore, some of them pointed to the inconsistency of the GRNs, or gene sets, when evaluated with small sets of regulatory data.

Recently, Larsen et al.^30^ studied *E. coli* GRNs and found that they are inconsistent when evaluated with gene expression data. The authors used a conservative sign consistency approach on a large microarray data compendium. Here, we analyzed the consistency of *C. glutamicum* GRN using a similar approach and also included RNA-seq gene expression data to obtain an exhaustive data compendium. In general, we assume that activations should increase the expression of the TGs when the TF is upregulated. Likewise, repressions should reduce the TGs’ expression, when the TF is also upregulated. Our results show a positive correlation in both cases, contradicting our current understanding of the role of TF regulation. The consistency model assessment indicates that the *C. glutamicum* GRN is even less consistent than random GRNs, implying both that additional research is needed to further refine GRNs and additional factors have to be considered to explain gene expression.

## Materials and Methods

### *C. glutamicum* GRN and gene expression data

The experimental GRN of *C. glutamicum* was downloaded from CoryneRegNet 7.0.^19^ The gene expression compendium was retrieved from Gene Expression Omnibus (GEO)^31^ and consisted of microarray and RNA-seq data with a total of 429 samples (see Supplementary Data S1 for more information about the datasets).

### Gene expression data normalization

All gene expression datasets were normalized using limma.^32^ Microarray data were background corrected before applying lowess (two-color microarrays) and quantile normalization. Similarly, RNA-seq data were quantile normalized using the voom method.^33^ Finally, to have all the data on the same scale, we combined and *z*-score normalized all gene expression data using the survJamda package.^34^ The *z*-normalized distribution of the expression data is given in Supplementary Figure S1.

### *In silico* data generation

The *in silico* GRN and gene expression data were generated using GeneNetWeaver.^35^ This software uses an experimental network as the model to create an *in silico* network with similar topology and to simulate gene expression data for the novel network.

### Inconsistency detection and assessment

We used the same method applied by Larsen et al.^30^ to assess *E. coli* GRN. In the first step, the method identifies genes that are up- or downregulated in each experiment (contrasts). This is performed by computing the contrasts as the difference between the expression of the reference and the case(s) in the experiment. Next, it uses a conservative sign consistency model similar to the one applied in COMA^27^ and BioQuali.^26^ For each contrast, the model labels the vertices as: upregulated, downregulated, or unchanged. The labels are attributed based on the expression differences between the control and each case in the contrast, and on a threshold _*t*_. Then the labels are compared with the role of the regulatory interactions in the GRN to determine the consistency. Finally, we compared the experimental network with random data by applying two perturbation methods. The first method shuffles the expression profiles and keeps the network topology, whereas the second shuffles the network topology and keeps the node degrees and gene expression profiles. For more details about this method, refer to the work from Larsen et al.^30^

### Statistical analyses

The mean correlation (*mc*) was calculated by first adding the correlation of each TF and TG/operon pair (*cPairs*), and then dividing the sum by the total number of pairs (*nPairs*). 
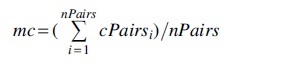


This same process was applied to compute the means for Pearson's and Spearman's correlation. We computed the mean of the global inconsistency load (*mGlobal*) of the two applied perturbation methods by adding the global inconsistency load (*global*) of each iteration and then dividing the sum by the number of iterations (*nIteration*). 
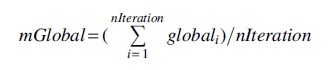


We determined the mean edge inconsistency (*mEdge*) through the sum of the number of inconsistencies of each TF and TG/operon pair (*nIncons*), which we then divided by the total number of pairs (*nPairs*). 
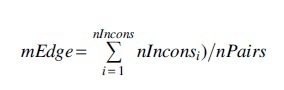


The significance of the comparison between the inconsistency load in contrasts with and without perturbation was computed using the Mann–Whitney *U*-test.^36^ We used the same test to compute the significance of the number of up- or downregulated genes in each contrast, with and without perturbation.

## Results

## Correlation between gene regulatory interactions and gene expression profiles

**FIG. 1.**
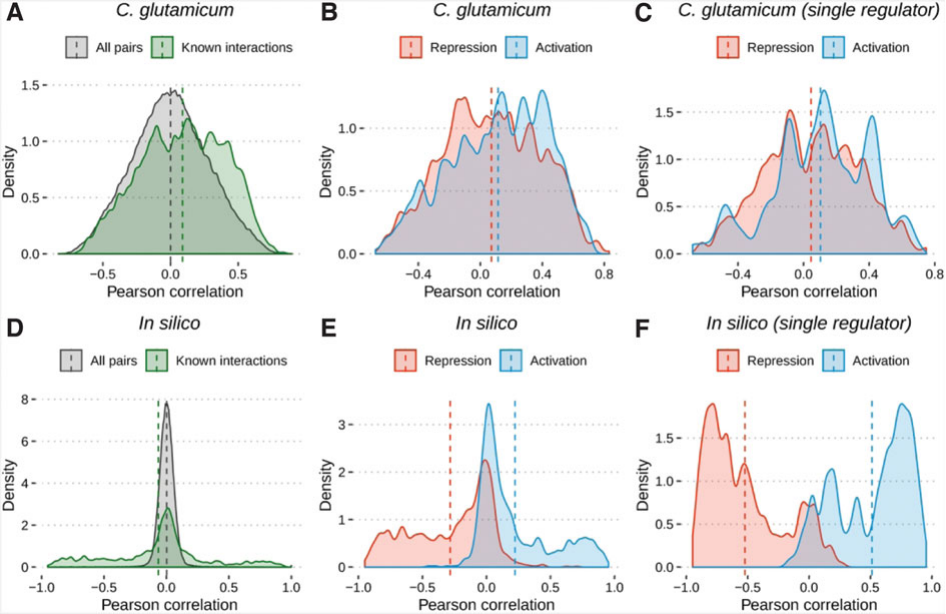
Distribution of Pearson correlation for TF and TG/operon pairs. Comparison between the correlation of all possible TF–TG pairs and all known TF–TG pairs. [**(A)** *Corynebacterium glutamicum* and **(D)** *in silico*]. Comparison between the correlation of known TF–TG pairs separated by interaction role: activation and repression [**(B)** *C. glutamicum* and **(E)** *in silico*]. Comparison between the correlation of known TF–TG pairs where each TG has only one regulator [**(C)** *C. glutamicum* and **(F)** *in silico*]. Dashed vertical lines show the mean correlation for each TF and TG/operon pair. TF, transcription factor; TG, target gene.

To assess the correlation between expression profiles of known regulatory interactions (e.g., a TF regulating a TG), we applied Pearson's correlation coefficient (Fig. 1). We analyzed the distribution of correlations between known TF–TG pairs and of all possible TF–TG pairs. The mean correlation of known TF–TG pairs is 0.09, whereas for all the possible TF–TG pairs it is −0.0003 (Fig. 1A). For activations, we expect a positive correlation (e.g., increased TF expression enhances TG expression), whereas repressions should result in a negative correlation (e.g., increased TF expression reduces TG expression). However, separately analyzing the distribution of the correlations of known TF–TG pairs by interaction role, we see a very low mean correlation in both cases: 0.11 for activating interactions and to 0.07 for repressing interactions. Complex regulations where multiple TFs control the same TG could influence the results observed in Figure 1B. Taking this into account, we also analyzed the TF–TG pairs where the TG is regulated by a single TF (Fig. 1C). Of interest, the results are similar (0.10 and 0.04 for activating and repressing interactions, respectively).

The same process was repeated using an *in silico* network and gene expression data to demonstrate the extent of the correlations expected from a GRN that is consistent with the observed expression data. The *in silico* data were generated based on the *C. glutamicum* GRN using GeneNetWeaver.^35^

The mean correlation of known interactions was −0.06 and the mean correlation of all possible TF–TG pairs was 0.002 (Fig. 1D). The correlation of the interactions (Fig. 1E) separated by the interaction role presented a distinct partition between them: the mean correlation was 0.22 and −0.28 for activation and repression interactions, respectively (Fig. 1E). Upon analyzing only the single regulators, an even stronger separation between them was noticed: mean 0.51 and −0.52 for activation and repression interactions, respectively (Fig. 1F).

### Consistency assessment between the regulatory networks and the expression profiles

**FIG. 2.**
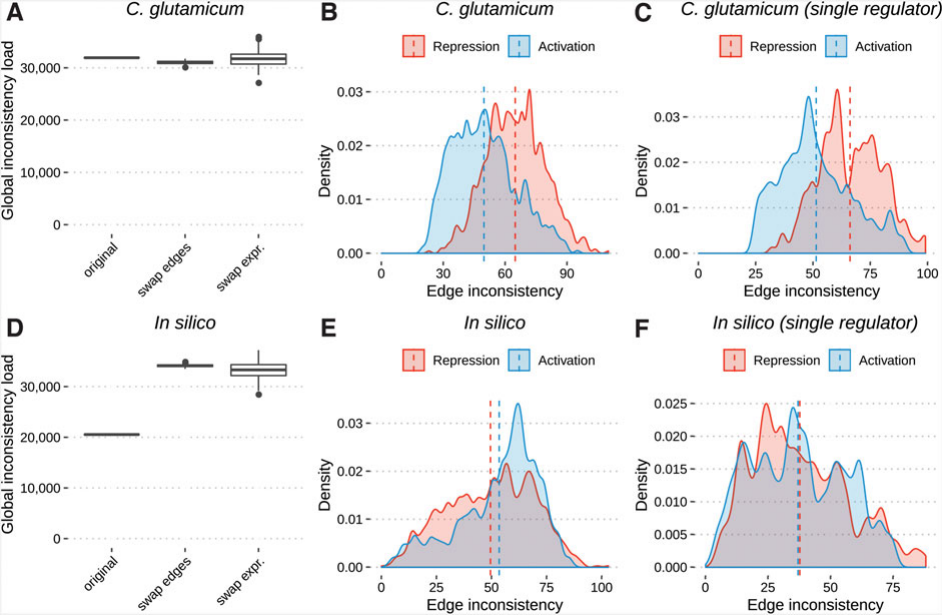
Evaluation of the inconsistency load in the GRN and perturbed GRN models. Comparison among the global inconsistency load (total number of inconsistent cases) in the GRNs with two random GRN models. The experiments were repeated 200 times for the random models [**(A)** *Corynebacterium glutamicum* and **(D)** *in silico*]. The edge inconsistency distribution split by interaction role: repression and activation [**(B)** *C. glutamicum* and **(E)** *in silico*]. The edge inconsistency distribution is split by role: repression and activation where a single TF regulates the TG/operon **(C, F)**. In [**(B, C)** *C. glutamicum*, **(E, F)** *in silico*] dashed vertical lines show the mean inconsistency for each pair TF and TG/operon. GRN, Gene Regulatory Network.

To assess the consistency between the GRN and gene expression, the model we applied makes use of a threshold. Our threshold considers any changes in the expression data (upregulation, downregulation, or unchanged) and assumes ∼50% of the contrasts to be up- or downregulated, as previously explained by Larsen et al.^30^ This resulted in a threshold of ±0.25 for the experimental data and ±0.82 for the *in silico* data. We compared the global inconsistency load of the experimental and *in silico* GRNs against randomly perturbed GRNs and expression profiles. The global inconsistency load (Fig. 2A) of the experimental network (31,922 inconsistencies) was higher than the mean of the perturbed data (31,030.30 and 31,733.00 inconsistencies in swapped edges and swapped expression profiles, respectively). These numbers indicate that the consistency between the experimental network and the expression data are not more significant than the consistency in the random networks. In contrast, the original *in silico* network (Fig. 2D) had fewer inconsistencies (20,538) than the mean of the perturbed data (34,133.40 and 33,180.00 in swapped edges and swapped expression profiles, respectively).

Analyzing the edge inconsistencies separated by the interaction role revealed that the number of inconsistencies was larger for repressions (mean 64.8) than for activations (mean 49.7; Fig. 2B). The analysis of single regulators presented similar results, but slightly larger means (mean 66.1 and 51.3 for repressions and activations, respectively) in both cases (Fig. 2C). For the *in silico* data, the inconsistencies separated by the interaction role (Fig. 2E) presented similar numbers in both cases (mean of 49.5 and 53.4 for repressions and activations, respectively). The results for single regulators (Fig. 2F) were smaller and even more similar (mean 37.7 and 37 for repressions and activations, respectively).

### Association between inconsistency load in contrast with and without perturbation

**FIG. 3.**
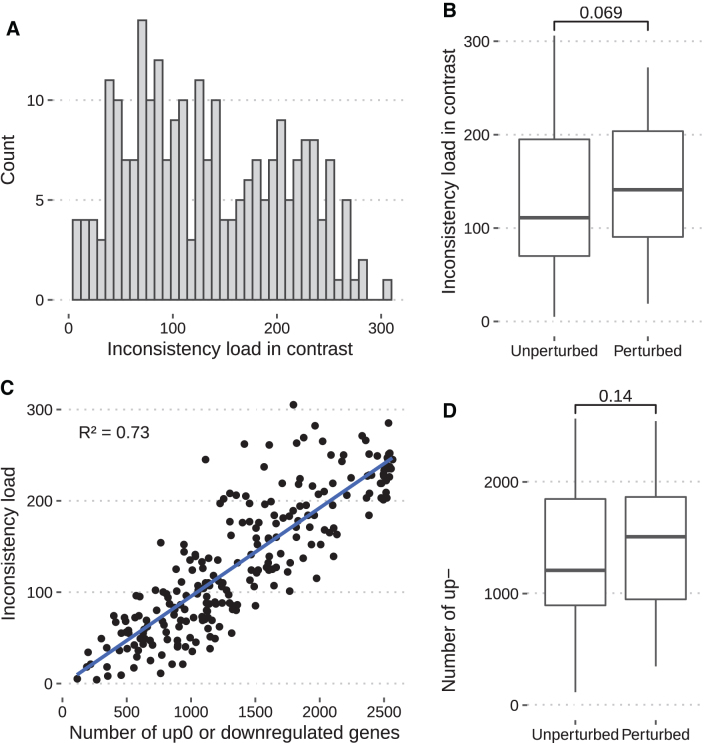
Evaluation of the inconsistency load of *Corynebacterium glutamicum* across contrasts. Distribution of the number of inconsistencies across the 239 contrasts **(A)**. Perturbations (e.g., stress conditions) increase the inconsistency load in contrasts when compared with nonperturbations **(B)**. Relationship between the number of deregulated genes (up or down) and inconsistency load in contrasts **(C)**. Comparison between the number of deregulated genes in the model for the contrasts with and without perturbation **(D)**. The *p*-values in **(B)** and **(D)** were computed using the Mann–Whitney *U*-test.

To check if perturbed experiments result in a higher level of inconsistencies, we analyzed the inconsistency load across the cases in each experiment, resulting in 239 contrasts. It resulted in a range from 5 to 306 inconsistencies with a mean of 133.56 (Fig. 3A). The number of inconsistencies was slightly more substantial in the contrasts with perturbed conditions than with the unperturbed ones (means of 145.31 and 127.88, respectively; Fig. 3B). Perturbed conditions in the experimental gene expression profiles include stress, overexpressed genes, knockout, and double-knockout genes. Genes considered to be up- or downregulated are associated with a higher level of inconsistencies in the network (Fig. 3C). Finally, a higher number of genes on average were considered up- or downregulated within the perturbed contrasts (Fig. 3D).

## Discussion

In this work, we applied microarray and RNA-seq data to investigate the widely accepted assumption in which changes in the expression of gene regulators affect the expression of their TGs. The regulation type (activation or repression) determines the effect of expression changes. Of interest, our results show that this is not the case for *C. glutamicum*, particularly at the transcriptional level. We found a positive mean correlation between the TFs and the TGs, even for repression interactions, which are supposed to have a negative correlation. When multiple TFs regulate the same TG, we cannot expect all regulations to be consistent. However, when analyzing the TGs that are regulated by a single regulator, a positive mean correlation between the TFs and the TGs was also observed. Larsen et al. found a similar behavior in the *E. coli* GRN^30^; considering it is more complete than *C. glutamicum* GRN,^37^ it is not surprising that *C. glutamicum* GRN is also not consistent with its expression data. Pearson's correlation analysis may not identify nonlinear relationships between the expression of TFs and TGs. We, thus, also applied Spearman's rank correlation (Supplementary Fig. S2) and observed a slightly positive interaction when analyzing, first, all known interactions at once (mean correlation of 0.09), then those split by interaction role (activations and repressions, mean correlation of 0.12 and 0.08, respectively) and, finally, for single regulators (mean correlation of 0.11 and 0.05 for activations and repressions, respectively). It demonstrates that nonlinearity does not affect our results.

The analysis of the global inconsistency load (Fig. 2A) shows that the experimental network is slightly more inconsistent than the perturbed ones. This may imply that the experimental GRN is not explained by the expression data retrieved from the GEO database. Because the chosen threshold could have influenced these results, we repeated the analysis using thresholds that consider ∼33% and ∼66% of the contrasts as up- or downregulated, as previously suggested by Larsen et al.,^30^ with no noticeable effect on the inconsistency between the GRN and the gene expression data (Supplementary Figs. S3–S6). Supplementary Figures S7–S9 demonstrate that network perturbation increases the global inconsistency of the in silico network while it has little effect on *C. glutamicum* GRN.

The high inconsistency between the *C. glutamicum* network and the expression data may be owing to technical or biological reasons. For example, the methods used to generate the GRNs from the experimental data may have performed poorly.^10,11,21,22^ Another explanation may be the unavailability of adequate time-series experimental data, which would reveal regulatory interactions at different time points.^38^ In addition, other regulatory mechanisms such as post-translational modifications,^39,40^ inactive conformation of the TFs and metabolites^23^ are neither identified by microarrays nor by RNA-seq data. Moreover, a low correlation between transcriptome and proteome data exists.^41,42^

## Conclusion

Our results corroborate previous studies that analyzed the consistency between *E. coli* GRN and gene expression data, and further showed that, when considering the static GRN, the networks are inconsistent.^23,25,30^ These results suggest that the traditional methods to reconstruct GRNs may not be able to fully represent the complexity of gene expression regulation. In the case of *C. glutamicum*, an accurate and consistent GRN is essential for the development of various robust strains that are required to meet its industrial demand in the production of biomolecules, such as amino acids.^43^ Although the accurate construction of *C. glutamicum* GRNs may provide an understanding of possible biosynthetic routes for these molecules, the inconsistency of GRNs and gene expression data suggest that extra caution should still prevail when using GRNs to elucidate probable amino acid biosynthetic routes in the biotechnology industry.

In our view, the incorporation of multi-omics time-series data and robust statistical approaches, as well as the performance of multiple perturbations within biological systems, are necessary to model the GRNs accurately. Moreover, a single GRN may not be adequate to capture the regulatory landscape across all possible conditions, requiring the development of condition-specific and dynamic GRNs in the future.

## Supplementary Material

Supplemental data

Supplemental data

Supplemental data

## Availability of Data and Materials

All data used in this work are publicly available on the GEO database (https://www.ncbi.nlm.nih.gov/geo). See Supplementary Table S1 in Supplementary Data S2 for more details.

## Abbreviations Used

GEO: Gene Expression Omnibus
GRNs: Gene Regulatory Networks
TFs: transcription factors
TGs: target genes

## References

[1] Land M, Hauser L, Jun SR, et al. Insights from 20 years of bacterial genome sequencing. Funct Integr Genomics. 2015;15:141–161 25722247 10.1007/s10142-015-0433-4 PMC4361730

[2] López-Maury L, Marguerat S., Bähler J. Tuning gene expression to changing environments: from rapid responses to evolutionary adaptation. Nat Rev Genet. 2008;9:583–593 18591982 10.1038/nrg2398

[3] Babu MM, Lang B, Aravind L. Methods to reconstruct and compare transcriptional regulatory networks. Methods Mol Biol. 2009;541:163–180 19381525 10.1007/978-1-59745-243-4_8

[4] Baumbach J, Brinkrolf K, Czaja LF, et al. CoryneRegNet: an ontology-based data warehouse of corynebacterial transcription factors and regulatory networks. BMC Genomics. 2006;7:24 16478536 10.1186/1471-2164-7-24 PMC1382212

[5] Schena M, Shalon D, Davis RW, et al. Quantitative monitoring of gene expression patterns with a complementary DNA microarray. Science. 1995;270:467–470 7569999 10.1126/science.270.5235.467

[6] Barski A, Cuddapah S, Cui K, et al. High-resolution profiling of histone methylations in the human genome. Cell. 2007;129:823–837 17512414 10.1016/j.cell.2007.05.009

[7] Nagalakshmi U, Wang Z, Waern K, et al. The transcriptional landscape of the yeast genome defined by RNA sequencing. Science. 2008;320:1344–1349 18451266 10.1126/science.1158441 PMC2951732

[8] Horak CE, Snyder M. ChIP-chip: a genomic approach for identifying transcription factor binding sites. Methods Enzymol. 2002;350:469–483 12073330 10.1016/s0076-6879(02)50979-4

[9] Bumgarner R. Overview of DNA microarrays: types, applications, and their future. Curr Protoc Mol Biol. 2013;Chapter 22:Unit 22.1 10.1002/0471142727.mb2201s101 PMC4011503 23288464

[10] Marbach D, Costello JC, Küffner R, et al. Wisdom of crowds for robust gene network inference. Nat Methods. 2012;9:796–804 22796662 10.1038/nmeth.2016 PMC3512113

[11] Madhamshettiwar PB, Maetschke SR, Davis MJ, et al. Gene regulatory network inference: evaluation and application to ovarian cancer allows the prioritization of drug targets. Genome Med. 2012;4:41 22548828 10.1186/gm340 PMC3506907

[12] De Smet R, Marchal K. Advantages and limitations of current network inference methods. Nat Rev Microbiol. 2010;8:717–729 20805835 10.1038/nrmicro2419

[13] Faure A, Naldi A, Chaouiya C, et al. Dynamical analysis of a generic Boolean model for the control of the mammalian cell cycle. Bioinformatics. 2006;22:e124–e131 16873462 10.1093/bioinformatics/btl210

[14] Lovrics A, Gao Y, Juhász B, et al. Boolean modelling reveals new regulatory connections between transcription factors orchestrating the development of the ventral spinal cord. PLoS One. 2014;9:e111430 25398016 10.1371/journal.pone.0111430 PMC4232242

[15] Friedman N, Linial M, Nachman I, Pe'er D. Using Bayesian networks to analyze expression data. Proceedings of the Fourth Annual International Conference on Computational Molecular Biology—RECOMB’00. Tokyo, Japan. April 2000. pp. 127–135 10.1089/106652700750050961 11108481

[16] Yu J, Smith VA, Wang PP, et al. Using Bayesian network inference algorithms to recover molecular genetic regulatory networks. Int Conf Syst Biol 2002;2002:ICSB02

[17] Santos-Zavaleta A, Salgado H, Gama-Castro S, et al. RegulonDB v 10.5: tackling challenges to unify classic and high throughput knowledge of gene regulation in *E*. coli K-12. Nucleic Acids Res. 2019;47:D212–D220 30395280 10.1093/nar/gky1077 PMC6324031

[18] Zhu B, Stülke J. SubtiWiki in 2018: from genes and proteins to functional network annotation of the model organism *Bacillus subtilis*. Nucleic Acids Res. 2018;46:D743–D748 29788229 10.1093/nar/gkx908 PMC5753275

[19] Parise MTD, Parise D, Kato RB, et al. CoryneRegNet 7, the reference database and analysis platform for corynebacterial gene regulatory networks. Sci Data. 2020;7:142 32393779 10.1038/s41597-020-0484-9 PMC7214426

[20] Escorcia-Rodríguez JM, Tauch A, Freyre-González JA. Abasy Atlas v2.2: the most comprehensive and up-to-date inventory of meta-curated, historical, bacterial regulatory networks, their completeness and system-level characterization. Comput Struct Biotechnol J. 2020;18:1228–1237 32542109 10.1016/j.csbj.2020.05.015 PMC7283102

[21] Chen S, Mar JC. Evaluating methods of inferring gene regulatory networks highlights their lack of performance for single cell gene expression data. BMC Bioinformatics. 2018;19:232 29914350 10.1186/s12859-018-2217-z PMC6006753

[22] Pratapa A, Jalihal AP, Law JN, et al. Benchmarking algorithms for gene regulatory network inference from single-cell transcriptomic data. Nat Methods. 2020:17:147–154 31907445 10.1038/s41592-019-0690-6 PMC7098173

[23] Gutiérrez-Ríos RM, Rosenblueth DA, Loza JA, et al. Regulatory network of *Escherichia coli*: consistency between literature knowledge and microarray profiles. Genome Res. 2003;13:2435–2443 14597655 10.1101/gr.1387003 PMC403762

[24] Siegel A, Radulescu O, Le Borgne M, et al. Qualitative analysis of the relation between DNA microarray data and behavioral models of regulation networks. Biosystems. 2006;84:153–174 16556482 10.1016/j.biosystems.2005.10.006

[25] Guziolowski C, Veber P, Le Borgne M, et al. Checking consistency between expression data and large scale regulatory networks: a case study. J Biol Phys Chem. 2007;7:37–43

[26] Guziolowski C, Bourdé A, Moreews F, et al. BioQuali Cytoscape plugin: analysing the global consistency of regulatory networks. BMC Genomics. 2009;10:244 19470162 10.1186/1471-2164-10-244 PMC2693143

[27] Baumbach J, Apeltsin L. Linking Cytoscape and the corynebacterial reference database CoryneRegNet. BMC Genomics. 2008;9:184 18426593 10.1186/1471-2164-9-184 PMC2375448

[28] Kittas A, Barozet A, Sereshti J, et al. CytoASP: a Cytoscape app for qualitative consistency reasoning, prediction and repair in biological networks. BMC Syst. Biol. 2015;9:34 26163265 10.1186/s12918-015-0179-6 PMC4499222

[29] Melas IN, Samaga R, Alexopoulos LG, et al. Detecting and removing inconsistencies between experimental data and signaling network topologies using integer linear programming on interaction graphs. PLoS Comput Biol. 2013;9:e1003204 24039561 10.1371/journal.pcbi.1003204 PMC3764019

[30] Larsen SJ, Röttger R, Schmidt HHHW, et al. *E. coli* gene regulatory networks are inconsistent with gene expression data. Nucleic Acids Res. 2019;47:85–92 30462289 10.1093/nar/gky1176 PMC6326786

[31] Barrett T, Wilhite SE, Ledoux P, et al. NCBI GEO: archive for functional genomics data sets—update. Nucleic Acids Res. 2013;41:D991–D995 23193258 10.1093/nar/gks1193 PMC3531084

[32] Ritchie ME, Phipson B, Wu D, et al. limma powers differential expression analyses for RNA-sequencing and microarray studies. Nucleic Acids Res. 2015;43:e47 25605792 10.1093/nar/gkv007 PMC4402510

[33] Law CW, Chen Y, Shi W, et al. voom: precision weights unlock linear model analysis tools for RNA-seq read counts. Genome Biol. 2014;15:R29 24485249 10.1186/gb-2014-15-2-r29 PMC4053721

[34] Yasrebi H. SurvJamda: an R package to predict patients’ survival and risk assessment using joint analysis of microarray gene expression data. Bioinformatics. 2011;27:1168–1169 21367873 10.1093/bioinformatics/btr103

[35] Schaffter T, Marbach D, Floreano D. GeneNetWeaver: *in silico* benchmark generation and performance profiling of network inference methods. Bioinformatics. 2011;27:2263–2270 21697125 10.1093/bioinformatics/btr373

[36] McKnight PE, Najab J. Mann-Whitney U Test. The Corsini Encyclopedia of Psychology. (Weiner IB, Craighead WE; eds). John Wiley & Sons, Inc., Hoboken, NJ. 2010

[37] Röttger R, Rückert U, Taubert J, et al. How little do we actually know? On the size of gene regulatory networks. IEEE/ACM Trans Comput Biol Bioinform. 2012;9:1293–1300 22585140 10.1109/TCBB.2012.71

[38] Yu H, Luscombe NM, Qian J, et al. Genomic analysis of gene expression relationships in transcriptional regulatory networks. Trends Genet. 2003;19:422–427 12902159 10.1016/S0168-9525(03)00175-6

[39] Hunter T, Karin M. The regulation of transcription by phosphorylation. Cell. 1992;70:375–387 1643656 10.1016/0092-8674(92)90162-6

[40] Filtz TM, Vogel WK, Leid M. Regulation of transcription factor activity by interconnected post-translational modifications. Trends Pharmacol Sci. 2014;35:76–85 24388790 10.1016/j.tips.2013.11.005 PMC3954851

[41] Maier T, Güell M, Serrano L. Correlation of mRNA and protein in complex biological samples. FEBS Lett. 2009;583:3966–3973 19850042 10.1016/j.febslet.2009.10.036

[42] Ghazalpour A, Bennett B, Petyuk VA, et al. Comparative analysis of proteome and transcriptome variation in mouse. PLoS Genet. 2011;7:e1001393 21695224 10.1371/journal.pgen.1001393 PMC3111477

[43] Ivanov K, Stoimenova A, Obreshkova D, et al. Biotechnology in the production of pharmaceutical industry ingredients: amino acids. Biotechnol Biotechnol Equipment. 2013;27:3620–3626

