# Supplementary material for: On the Consistency between Gene Expression and the Gene Regulatory Network of Corynebacterium glutamicum
Source: Netw Syst Med. 2021 Mar 8;4(1):51–9. doi: 10.1089/nsm.2020.0014 (PMC8006670; doi:10.1089/nsm.2020.0014)
Supplement: Supplemental data [file Supp_DataS1.docx]

**S1 Gene expression data**

A compendium combining gene expression from microarray and RNA-seq data was retrieved from GEO database.^1^ The microarray data was quantified using the GenePix platform. The RNASeq data was obtained from the Illumina platform. The used datasets are presented in Supplementary Table S1.

**References**

1. [Barrett T, Wilhite SE, Ledoux P, et al. NCBI GEO: archive for functional genomics data sets--update. Nucleic Acids Res. 2013;41:D991–5.](http://paperpile.com/b/d3FgTO/l7zNT)
