# Supplementary material for: On the Consistency between Gene Expression and the Gene Regulatory Network of Corynebacterium glutamicum
Source: Netw Syst Med. 2021 Mar 8;4(1):51–9. doi: 10.1089/nsm.2020.0014 (PMC8006670; doi:10.1089/nsm.2020.0014)
Supplement: Supplemental data [file Supp_DataS2.zip › Supp_Fig1.docx]

**
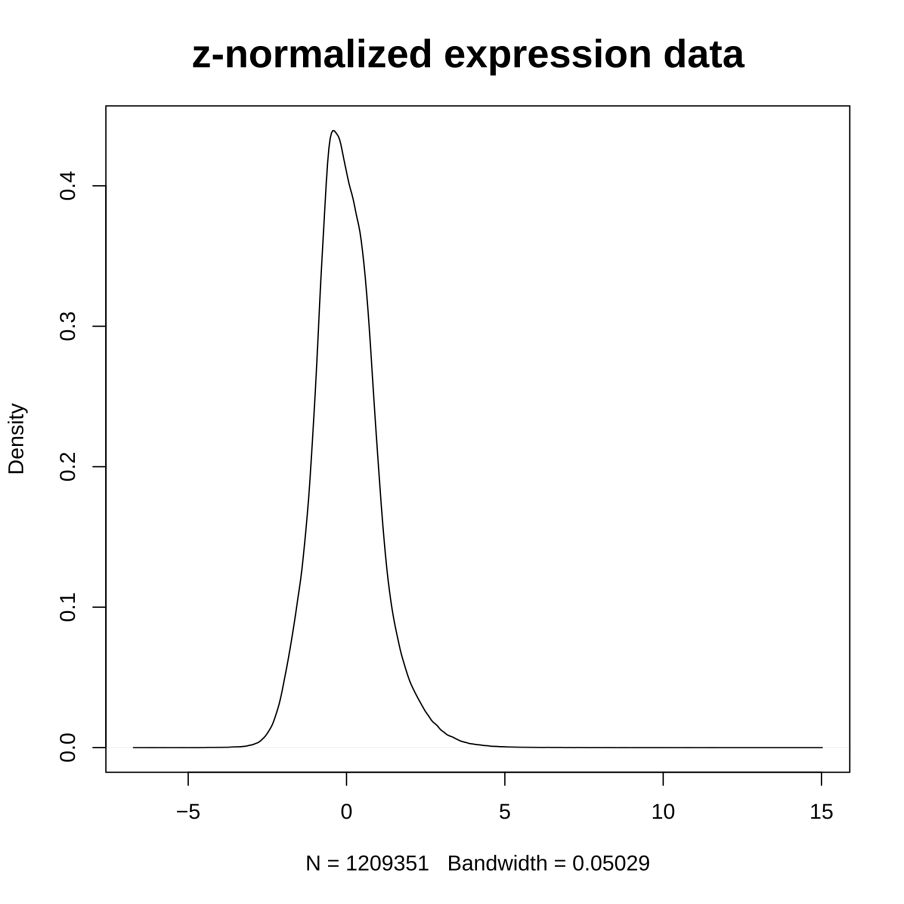
**

**Figure S1 - The z-score normalized gene expression data distribution.** The z-score describes the relationship of the values to the mean of the group, in terms of standard deviations.
