# Supplementary material for: On the Consistency between Gene Expression and the Gene Regulatory Network of Corynebacterium glutamicum
Source: Netw Syst Med. 2021 Mar 8;4(1):51–9. doi: 10.1089/nsm.2020.0014 (PMC8006670; doi:10.1089/nsm.2020.0014)
Supplement: Supplemental data [file Supp_DataS2.zip › Supp_Fig2.docx]

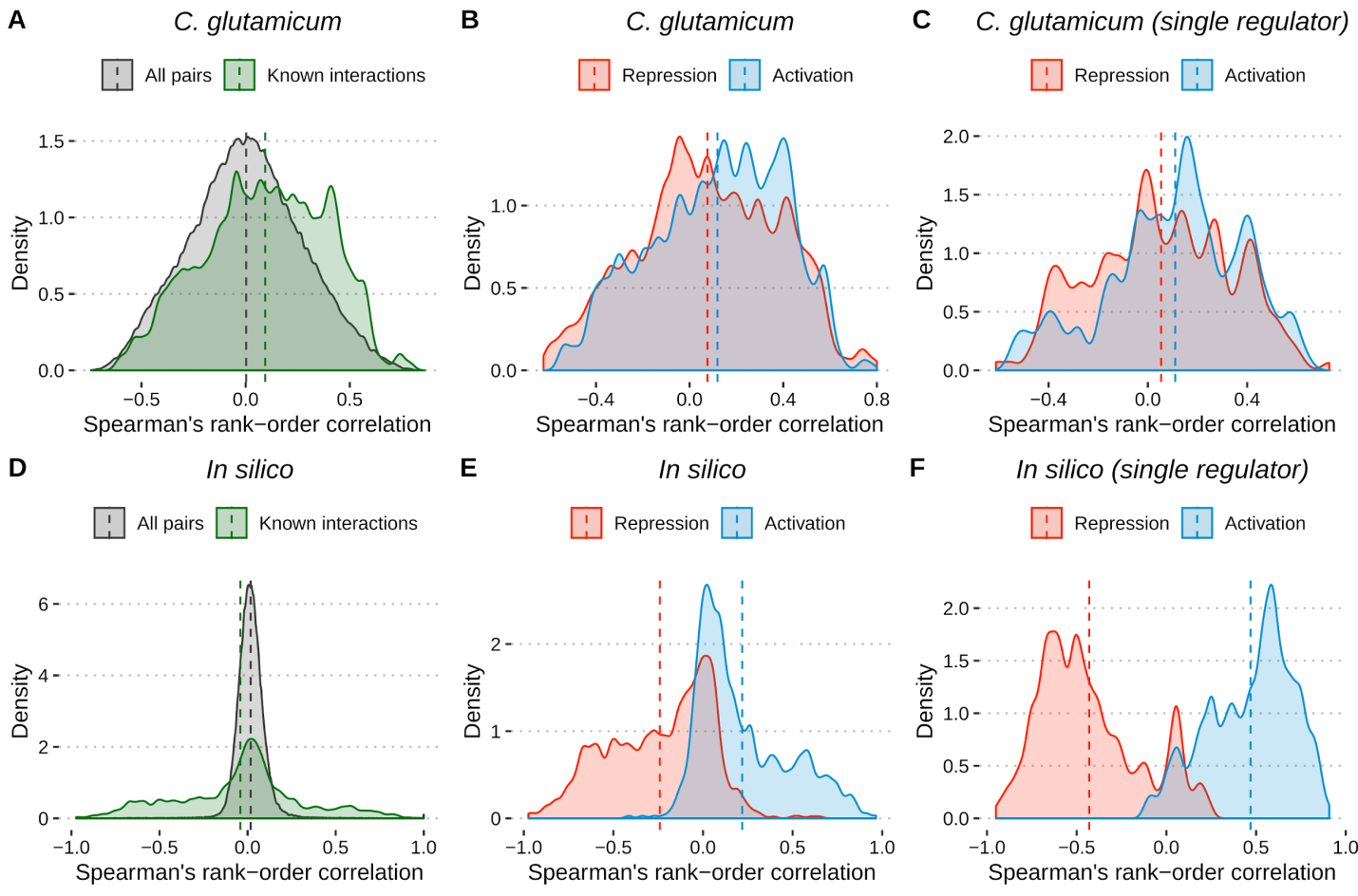


**Figure S2 - Distribution of Spearman's rank-correlation for TF and target gene/operon pairs.** Comparison between the correlation of all possible TF-TG pairs and all known TF-TG pairs (A [*C. glutamicum*] and D [*in silico*]). Comparison between the correlation of known TF-TG pairs separated by interaction role: activation and repression (B [*C. glutamicum*] and E [*in silico*]). Comparison between the correlation of known TF-TG pairs where each TG has only one regulator (C [*C. glutamicum*] and F [*in silico*]). Dashed vertical lines show the mean correlation for each TF and TG/operon pair.
