# Supplementary material for: On the Consistency between Gene Expression and the Gene Regulatory Network of Corynebacterium glutamicum
Source: Netw Syst Med. 2021 Mar 8;4(1):51–9. doi: 10.1089/nsm.2020.0014 (PMC8006670; doi:10.1089/nsm.2020.0014)
Supplement: Supplemental data [file Supp_DataS2.zip › Supp_Fig3.docx]

**
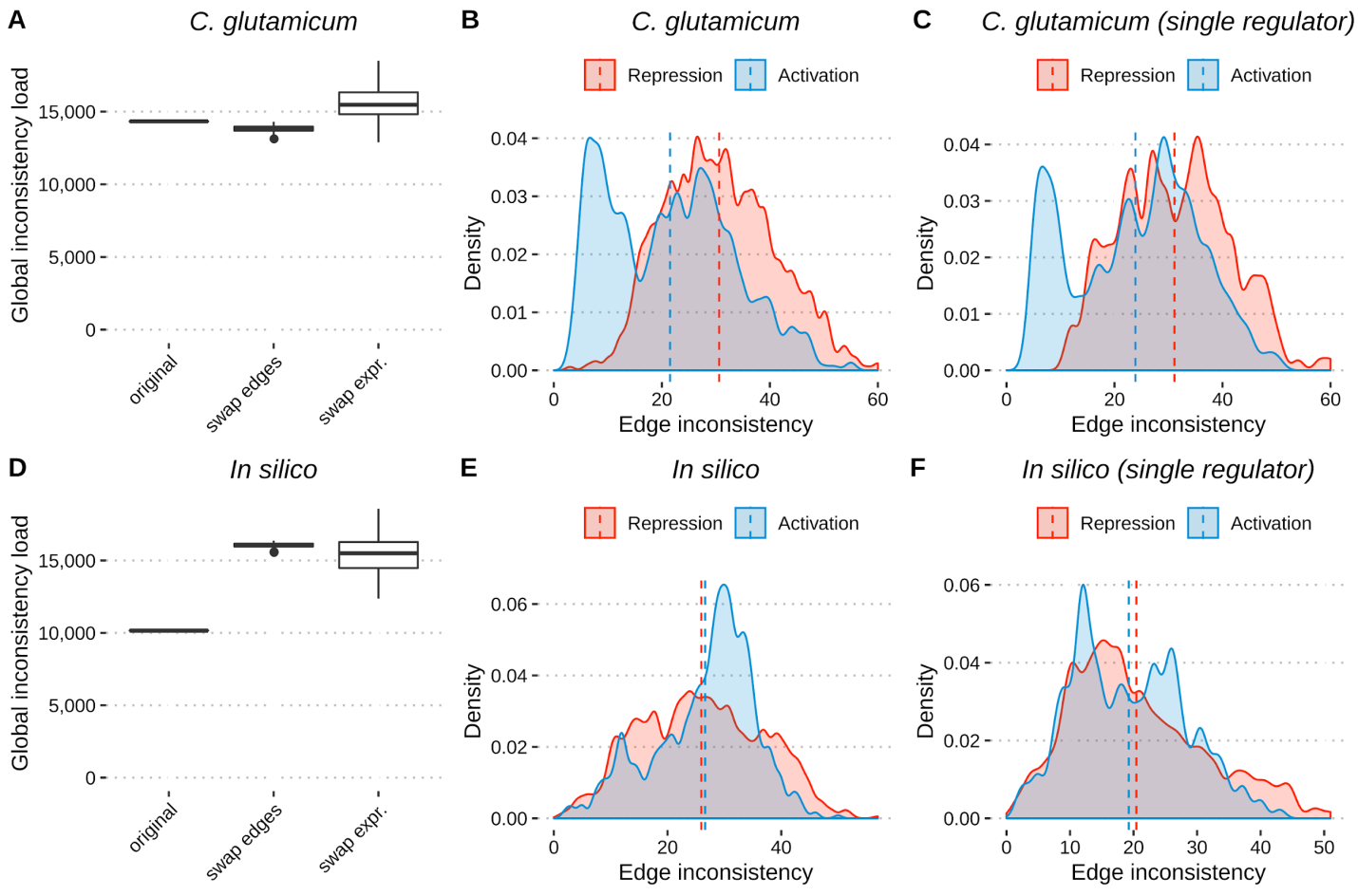
**

**Figure S3 - Evaluation of the inconsistency load in the GRN and perturbed GRN models.** Here we chose a threshold that considers 33% of the contrasts values as up- or downregulated. Comparison among the global inconsistency load (total number of inconsistent cases) in the GRNs with two random GRN models. The experiments were repeated 200 times for the random models (A [*C. glutamicum*] and D [*in silico*]). The edge inconsistency distribution split by interaction role: repression and activation (B [*C. glutamicum*] and E [*in silico*]). The edge inconsistency distribution is split by role: repression and activation where a single TF regulates the TG/operon (C [*C. glutamicum*] and F [*in silico*]). In (B, C [*C. glutamicum*], E and F [*in silico*]) dashed vertical lines show the mean inconsistency for each TF and TG/operon pair.
