# Supplementary material for: On the Consistency between Gene Expression and the Gene Regulatory Network of Corynebacterium glutamicum
Source: Netw Syst Med. 2021 Mar 8;4(1):51–9. doi: 10.1089/nsm.2020.0014 (PMC8006670; doi:10.1089/nsm.2020.0014)
Supplement: Supplemental data [file Supp_DataS2.zip › Supp_Fig6.docx]

**
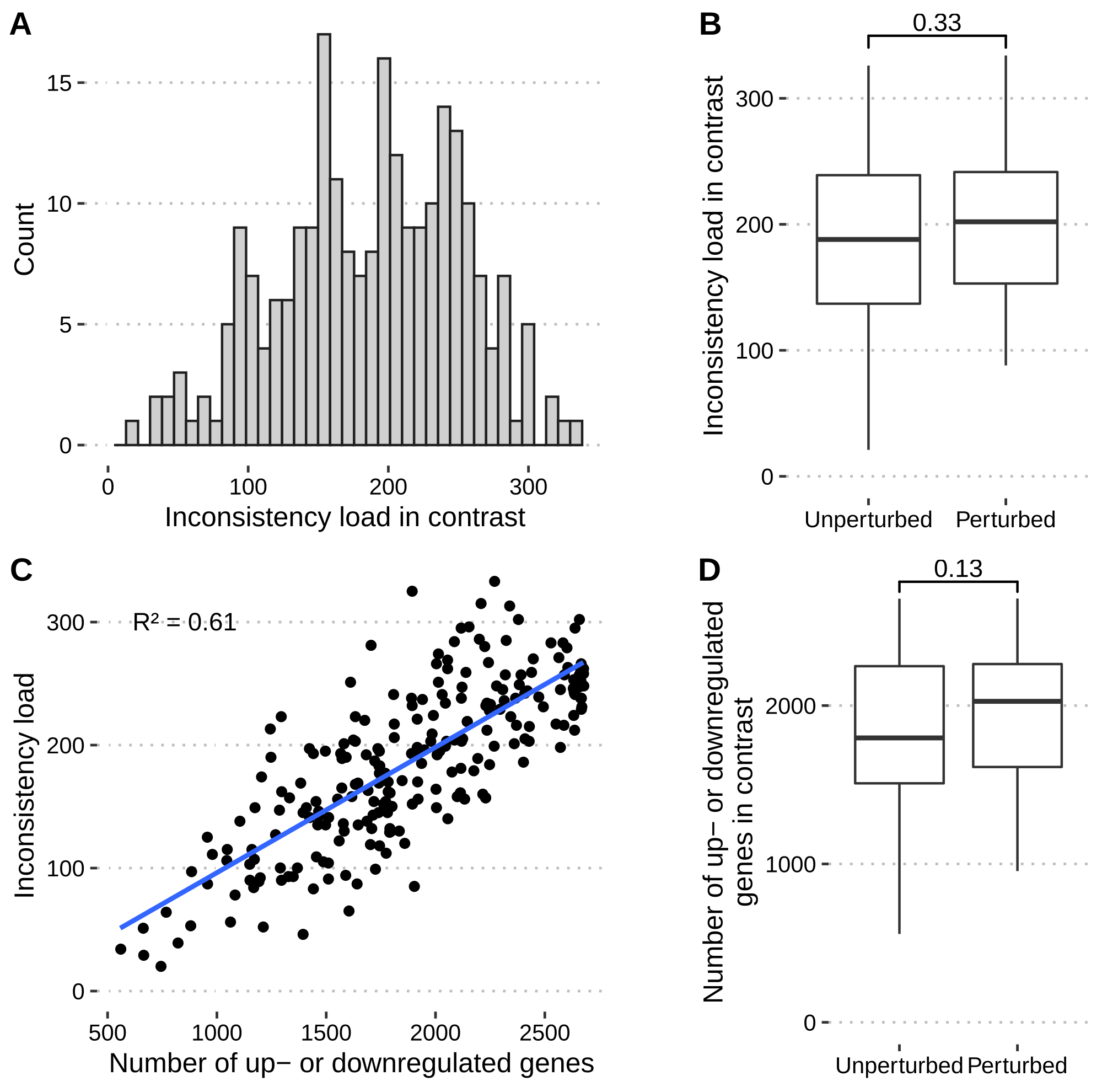
**

**Figure S6 - Evaluation of the inconsistency load of *C. glutamicum* across contrasts.** Here we chose a threshold that considers 66% of the contrasts values as up- or downregulated. Number of inconsistencies distribution inconsistency load distribution across the 239 contrasts (A). Perturbations (e.g. stress conditions) increase the inconsistency load in contrasts when compared to non-perturbations (B). Relationship between the number of deregulated genes (up or down) and inconsistency load in contrasts (C). Comparison between the number of deregulated genes in the model for in contrasts with and without perturbation (D). The p-values in (B) and (D) were computed using the Mann–Whitney U-test.
