# Supplementary material for: On the Consistency between Gene Expression and the Gene Regulatory Network of Corynebacterium glutamicum
Source: Netw Syst Med. 2021 Mar 8;4(1):51–9. doi: 10.1089/nsm.2020.0014 (PMC8006670; doi:10.1089/nsm.2020.0014)
Supplement: Supplemental data [file Supp_DataS2.zip › Supp_Fig7.docx]

**
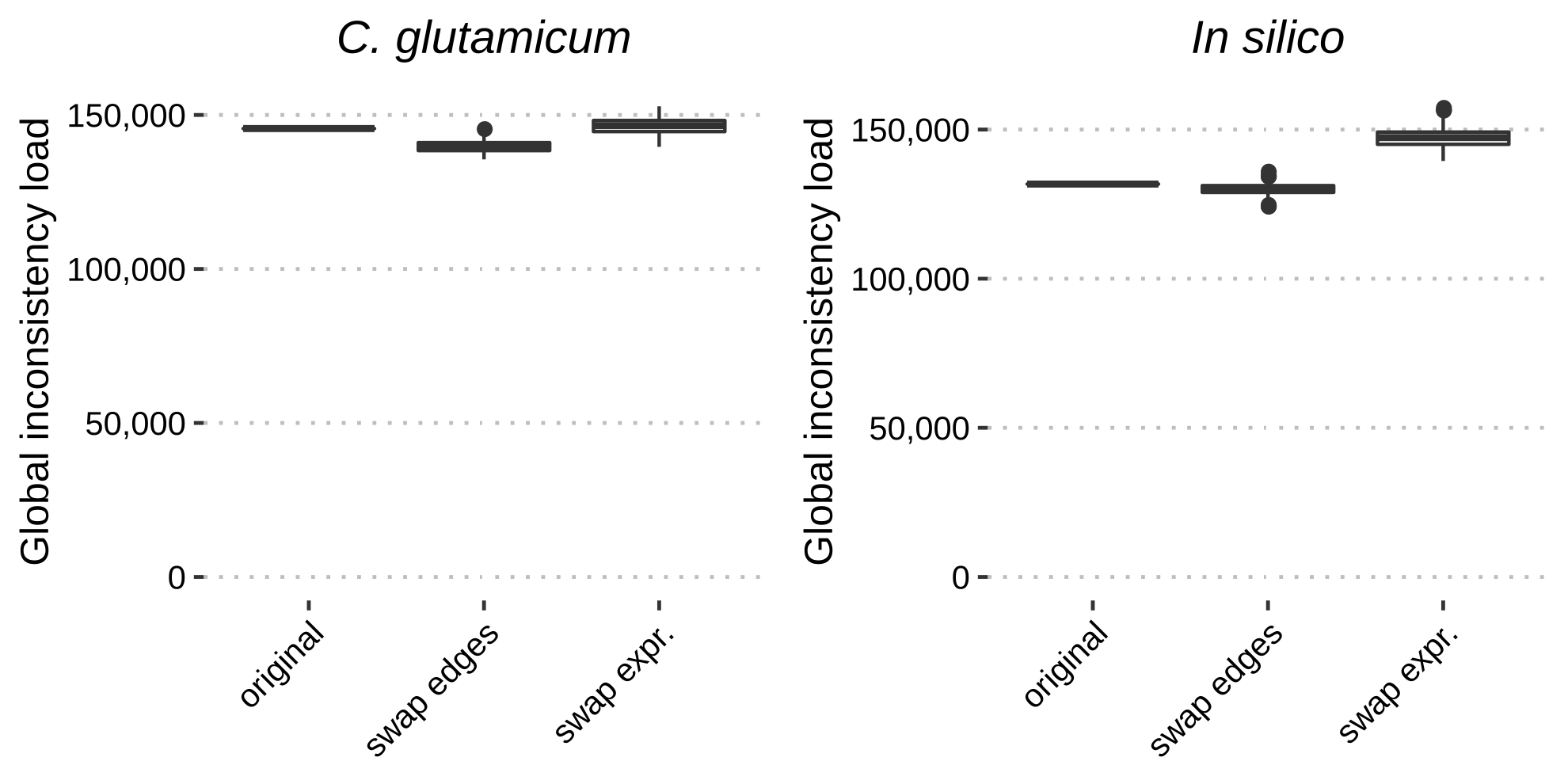
Figure S7 - Comparisons of the global inconsistency load of the GRN against the two random network models.** In this analysis, only data above the 13th percentile were considered. The experiments were repeated 200 times for the random models.
