# Supplementary material for: On the Consistency between Gene Expression and the Gene Regulatory Network of Corynebacterium glutamicum
Source: Netw Syst Med. 2021 Mar 8;4(1):51–9. doi: 10.1089/nsm.2020.0014 (PMC8006670; doi:10.1089/nsm.2020.0014)
Supplement: Supplemental data [file Supp_Table1.docx]

Supplementary Table S1 - Datasets

**Author Year Reference Plataform Type Dataset Sample number Condition**

Rehm et. al. 2010

Rehm N, Georgi T, Hiery E, Degner U et al. L-

Glutamine as a nitrogen source for Corynebacterium glutamicum: derepression of the AmtR regulon and implications for nitrogen sensing. Microbiology 2010 Oct;156(Pt 10):3180-

3193. PMID: 20656783 GPL9860 Microarray GSE19779 GSM493936 Ammonia x Glutamine

Rehm et. al. 2010

Rehm N, Georgi T, Hiery E, Degner U et al. L-

Glutamine as a nitrogen source for Corynebacterium glutamicum: derepression of the AmtR regulon and implications for nitrogen sensing. Microbiology 2010 Oct;156(Pt 10):3180-

3193. PMID: 20656783 GPL9860 Microarray GSE19779 GSM493937 Glutamine x Ammonia

Rehm et. al. 2010

Rehm N, Georgi T, Hiery E, Degner U et al. L-

Glutamine as a nitrogen source for Corynebacterium glutamicum: derepression of the AmtR regulon and implications for nitrogen sensing. Microbiology 2010 Oct;156(Pt 10):3180-

3193. PMID: 20656783 GPL9860 Microarray GSE19779 GSM493938 Ammonia x Glutamine

Frunzke et. al. 2010

Frunzke J, Gätgens C, Brocker M, Bott M. Control of heme homeostasis in Corynebacterium glutamicum by the two-component system HrrSA. J Bacteriol 2011 Mar;193(5):1212-21.

PMID: 21217007 GPL9860 Microarray GSE26122 GSM641539 FeSO4 x heme

Frunzke et. al. 2010

Frunzke J, Gätgens C, Brocker M, Bott M. Control of heme homeostasis in Corynebacterium glutamicum by the two-component system HrrSA. J Bacteriol 2011 Mar;193(5):1212-21.

PMID: 21217007 GPL9860 Microarray GSE26122 GSM641540 FeSO4 x heme

Frunzke et. al. 2010

Frunzke J, Gätgens C, Brocker M, Bott M. Control of heme homeostasis in Corynebacterium glutamicum by the two-component system HrrSA. J Bacteriol 2011 Mar;193(5):1212-21.

PMID: 21217007 GPL9860 Microarray GSE26122 GSM641541 FeSO4 x heme

Frunzke et. al. 2010

Frunzke J, Gätgens C, Brocker M, Bott M. Control of heme homeostasis in Corynebacterium glutamicum by the two-component system HrrSA. J Bacteriol 2011 Mar;193(5):1212-21.

PMID: 21217007 GPL9860 Microarray GSE26122 GSM641542 WT x DhrrA_FeSO4

Frunzke et. al. 2010

Frunzke J, Gätgens C, Brocker M, Bott M. Control of heme homeostasis in Corynebacterium glutamicum by the two-component system HrrSA. J Bacteriol 2011 Mar;193(5):1212-21.

PMID: 21217007 GPL9860 Microarray GSE26122 GSM641543 WT x DhrrA_FeSO4

Frunzke et. al. 2010

Frunzke J, Gätgens C, Brocker M, Bott M. Control of heme homeostasis in Corynebacterium glutamicum by the two-component system HrrSA. J Bacteriol 2011 Mar;193(5):1212-21.

PMID: 21217007 GPL9860 Microarray GSE26122 GSM641544 WT x DhrrA_FeSO4

Frunzke et. al. 2010

Frunzke J, Gätgens C, Brocker M, Bott M. Control of heme homeostasis in Corynebacterium glutamicum by the two-component system HrrSA. J Bacteriol 2011 Mar;193(5):1212-21.

PMID: 21217007 GPL9860 Microarray GSE26122 GSM641545 WT x DhrrA_heme

Frunzke et. al. 2010

Frunzke J, Gätgens C, Brocker M, Bott M. Control of heme homeostasis in Corynebacterium glutamicum by the two-component system HrrSA. J Bacteriol 2011 Mar;193(5):1212-21.

PMID: 21217007 GPL9860 Microarray GSE26122 GSM641546 WT x DhrrA_heme

Frunzke et. al. 2010

Frunzke J, Gätgens C, Brocker M, Bott M. Control of heme homeostasis in Corynebacterium glutamicum by the two-component system HrrSA. J Bacteriol 2011 Mar;193(5):1212-21.

PMID: 21217007 GPL9860 Microarray GSE26122 GSM641547 WT x DhrrA_heme

Schelder et. al. 2011

Schelder S, Zaade D, Litsanov B, Bott M et al. The two-component signal transduction system CopRS of Corynebacterium glutamicum is required for adaptation to copper-excess stress.

PLoS One 2011;6(7):e22143. PMID: 21799779 GPL9860 Microarray GSE27510 GSM679569 21.25 uM copper x 1.25 µM copper

Schelder et. al. 2011

Schelder S, Zaade D, Litsanov B, Bott M et al. The two-component signal transduction system CopRS of Corynebacterium glutamicum is required for adaptation to copper-excess stress.

PLoS One 2011;6(7):e22143. PMID: 21799779 GPL9860 Microarray GSE27510 GSM679574 21.25 uM copper x 1.25 µM copper

Schelder et. al. 2011

Schelder S, Zaade D, Litsanov B, Bott M et al. The two-component signal transduction system CopRS of Corynebacterium glutamicum is required for adaptation to copper-excess stress.

PLoS One 2011;6(7):e22143. PMID: 21799779 GPL9860 Microarray GSE27510 GSM679578 21.25 uM copper x 1.25 µM copper

Schelder et. al. 2011

Schelder S, Zaade D, Litsanov B, Bott M et al. The two-component signal transduction system CopRS of Corynebacterium glutamicum is required for adaptation to copper-excess stress.

PLoS One 2011;6(7):e22143. PMID: 21799779 GPL9860 Microarray GSE27510 GSM679580 21.25 uM copper x Delta copRS

Schelder et. al. 2011

Schelder S, Zaade D, Litsanov B, Bott M et al. The two-component signal transduction system CopRS of Corynebacterium glutamicum is required for adaptation to copper-excess stress.

| Schelder et. al. | 2011 | Schelder S, Zaade D, Litsanov B, Bott M et al. The two-component signal transduction system CopRS of Corynebacterium glutamicum is required for adaptation to copper-excess stress.  PLoS One 2011;6(7):e22143. PMID: 21799779 GPL9860 | |  | GSE27510 | GSM679586 | 21.25 uM copper x Delta copRS |
| --- | --- | --- | --- | --- | --- | --- | --- |
| Schelder et. al. | 2011 | Schelder S, Zaade D, Litsanov B, Bott M et al. The two-component signal transduction system CopRS of Corynebacterium glutamicum is required for adaptation to copper-excess stress.  PLoS One 2011;6(7):e22143. PMID: 21799779 GPL9860 | | Microarray | GSE27510 | GSM679588 | Delta copRS x 21.25 uM copper |
| Kleine et. al. | 2017 | Kleine B, Chattopadhyay A, Polen T, Pinto D et al. The three-component system EsrISR regulates a cell envelope stress response in Corynebacterium  glutamicum. Mol Microbiol2017 Dec;106(5):719-  741. PMID: 28922502 GPL9860 | | Microarray | GSE97961 | GSM2583853 | wt x delta_cgtSR7 |
| Kleine et. al. | 2017 | Kleine B, Chattopadhyay A, Polen T, Pinto D et al. The three-component system EsrISR regulates a cell envelope stress response in Corynebacterium  glutamicum. Mol Microbiol2017 Dec;106(5):719-  741. PMID: 28922502 GPL9860 | | Microarray | GSE97961 | GSM2583854 | delta_cgtSR7 x wt |
| Kleine et. al. | 2017 | Kleine B, Chattopadhyay A, Polen T, Pinto D et al. The three-component system EsrISR regulates a cell envelope stress response in Corynebacterium  glutamicum. Mol Microbiol2017 Dec;106(5):719-  741. PMID: 28922502 GPL9860 | | Microarray | GSE97961 | GSM2583855 | wt x delta_cgtSR7 |
| Kleine et. al. | 2017 | Kleine B, Chattopadhyay A, Polen T, Pinto D et al. The three-component system EsrISR regulates a cell envelope stress response in Corynebacterium  glutamicum. Mol Microbiol2017 Dec;106(5):719-  741. PMID: 28922502 GPL9860 | | Microarray | GSE97961 | GSM2583856 | wt x delta_cg0706 |
| Kleine et. al. | 2017 | Kleine B, Chattopadhyay A, Polen T, Pinto D et al. The three-component system EsrISR regulates a cell envelope stress response in Corynebacterium  glutamicum. Mol Microbiol2017 Dec;106(5):719-  741. PMID: 28922502 GPL9860 | | Microarray | GSE97961 | GSM2583857 | delta_cg0706 x wt |
| Kleine et. al. | 2017 | Kleine B, Chattopadhyay A, Polen T, Pinto D et al. The three-component system EsrISR regulates a cell envelope stress response in Corynebacterium  glutamicum. Mol Microbiol2017 Dec;106(5):719-  741. PMID: 28922502 GPL9860 | | Microarray | GSE97961 | GSM2583858 | wt x delta_cg0706 |
| Kleine et. al. | 2017 | Kleine B, Chattopadhyay A, Polen T, Pinto D et al. The three-component system EsrISR regulates a cell envelope stress response in Corynebacterium  glutamicum. Mol Microbiol2017 Dec;106(5):719-  741. PMID: 28922502 GPL9860 | | Microarray | GSE97961 | GSM2583859 | wt x bacitracin |
| Kleine et. al. | 2017 | Kleine B, Chattopadhyay A, Polen T, Pinto D et al. The three-component system EsrISR regulates a cell envelope stress response in Corynebacterium  glutamicum. Mol Microbiol2017 Dec;106(5):719-  741. PMID: 28922502 GPL9860 | | Microarray | GSE97961 | GSM2583860 | bacitracin x wt |
| Kleine et. al. | 2017 | Kleine B, Chattopadhyay A, Polen T, Pinto D et al. The three-component system EsrISR regulates a cell envelope stress response in Corynebacterium | | Microarray | GSE97961 | GSM2583861 | wt x bacitracin |
|  |  | glutamicum. Mol Microbiol2017 Dec;106(5):719741. PMID: 28922502 | GPL9860 |  |  |  |  |
| Wolf et. al. | 2020 | Wolf N, Bussmann M, Koch-Koerfges A,  Katcharava N et al. Molecular Basis of Growth  Inhibition by Acetate of an Adenylate CyclaseDeficient Mutant of <i>Corynebacterium glutamicum</i>. Front Microbiol 2020;11:87.  PMID: 32117117 | GPL9860 | Microarray | GSE140408 | GSM4160795 | wt x Δcg0375 |
| Wolf et. al. | 2020 | Wolf N, Bussmann M, Koch-Koerfges A,  Katcharava N et al. Molecular Basis of Growth  Inhibition by Acetate of an Adenylate CyclaseDeficient Mutant of <i>Corynebacterium glutamicum</i>. Front Microbiol 2020;11:87.  PMID: 32117117 | GPL9860 | Microarray | GSE140408 | GSM4160794 | Δcg0375 x wt |
| Wolf et. al. | 2020 | Wolf N, Bussmann M, Koch-Koerfges A,  Katcharava N et al. Molecular Basis of Growth  Inhibition by Acetate of an Adenylate CyclaseDeficient Mutant of <i>Corynebacterium glutamicum</i>. Front Microbiol 2020;11:87.  PMID: 32117117 | GPL9860 | Microarray | GSE140408 | GSM4160796 | wt x Δcg0375 |
| Jurischka et. al. | 2020 | Jurischka S, Bida A, Dohmen-Olma D, Kleine B et al. A secretion biosensor for monitoring Secdependent protein export in Corynebacterium glutamicum. Microb Cell Fact 2020 Jan  21;19(1):11. PMID: 31964372 | GPL9860 | Microarray | GSE140735 | GSM4182913 | wt x AmyE_noSP_induced |

PLoS One 2011;6(7):e22143. PMID: 21799779 GPL9860 Microarray GSE27510 GSM679582 21.25 uM copper x Delta copRS Schelder et. al. 2011

Schelder S, Zaade D, Litsanov B, Bott M et al. The two-component signal transduction system CopRS of Corynebacterium glutamicum is required for adaptation to copper-excess stress.

PLoS One 2011;6(7):e22143. PMID: 21799779 GPL9860 GSE27510 GSM679584 21.25 uM copper x Delta copRS

Jurischka et. al. 2020

Jurischka S, Bida A, Dohmen-Olma D, Kleine B et al. A secretion biosensor for monitoring Secdependent protein export in Corynebacterium glutamicum. Microb Cell Fact 2020 Jan

| Jurischka et. al. | 2020 | Jurischka S, Bida A, Dohmen-Olma D, Kleine B et al. A secretion biosensor for monitoring Secdependent protein export in Corynebacterium glutamicum. Microb Cell Fact 2020 Jan  21;19(1):11. PMID: 31964372 | GPL9860 |  | GSE140735 | GSM4182916 | wt x AmyE_induced |
| --- | --- | --- | --- | --- | --- | --- | --- |
| Jurischka et. al. | 2020 | Jurischka S, Bida A, Dohmen-Olma D, Kleine B et al. A secretion biosensor for monitoring Secdependent protein export in Corynebacterium glutamicum. Microb Cell Fact 2020 Jan  21;19(1):11. PMID: 31964372 | GPL9860 | Microarray | GSE140735 | GSM4182917 | wt x AmyE_induced |
| Jurischka et. al. | 2020 | Jurischka S, Bida A, Dohmen-Olma D, Kleine B et al. A secretion biosensor for monitoring Secdependent protein export in Corynebacterium glutamicum. Microb Cell Fact 2020 Jan  21;19(1):11. PMID: 31964372 | GPL9860 | Microarray | GSE140735 | GSM4182918 | wt x AmyE_induced |
| Jurischka et. al. | 2020 | Jurischka S, Bida A, Dohmen-Olma D, Kleine B et al. A secretion biosensor for monitoring Secdependent protein export in Corynebacterium glutamicum. Microb Cell Fact 2020 Jan  21;19(1):11. PMID: 31964372 | GPL9860 | Microarray | GSE140735 | GSM4182919 | wt x WT_NprECut_induced |
| Jurischka et. al. | 2020 | Jurischka S, Bida A, Dohmen-Olma D, Kleine B et al. A secretion biosensor for monitoring Secdependent protein export in Corynebacterium glutamicum. Microb Cell Fact 2020 Jan  21;19(1):11. PMID: 31964372 | GPL9860 | Microarray | GSE140735 | GSM4182920 | wt x WT_NprECut_induced |
| Jurischka et. al. | 2020 | Jurischka S, Bida A, Dohmen-Olma D, Kleine B et al. A secretion biosensor for monitoring Secdependent protein export in Corynebacterium glutamicum. Microb Cell Fact 2020 Jan  21;19(1):11. PMID: 31964372 | GPL9860 | Microarray | GSE140735 | GSM4182921 | WT_NprECut_induced x wt |
| Hyeon et. al. | 2011 | Hyeon JE, Kang DH, Kim YI, You SK et al. GntRtype transcriptional regulator PckR negatively regulates the expression of  phosphoenolpyruvate carboxykinase in  Corynebacterium glutamicum. J Bacteriol 2012  May;194(9):2181-8. PMID: 22366416 | GPL14656 | Microarray | GSE32573 | GSM807328 | Wild-type x Δcg0196 |
| Park et. al. | 2015 | Park, H.-S., Um, Y., Sim, S.J., Lee, S.Y., *Woo,  H.M., Transcriptomic analysis of  Corynebacterium glutamicum to overcome the toxicity of furfural present in hydrolysates. |  | Microarray | GSE55516 | GSM1338517 | wt x Furfural(6.5mM) |
|  |  | Process Biochemistry. Volume 50, Issue 3, March  2015, Pages 347-356  http://dx.doi.org/10.1016/j.procbio.2014.11.014 GPL14656 | |  |  |  |  |
| Park et. al. | 2015 | Park, H.-S., Um, Y., Sim, S.J., Lee, S.Y., *Woo,  H.M., Transcriptomic analysis of  Corynebacterium glutamicum to overcome the toxicity of furfural present in hydrolysates.  Process Biochemistry. Volume 50, Issue 3, March  2015, Pages 347-356  http://dx.doi.org/10.1016/j.procbio.2014.11.014 GPL14656 | | Microarray | GSE55516 | GSM1338518 | wt x Furfural(6.5mM) |
| Park et. al. | 2015 | Park, H.-S., Um, Y., Sim, S.J., Lee, S.Y., *Woo, H.M., Transcriptomic analysis of  Corynebacterium glutamicum to overcome the toxicity of furfural present in hydrolysates.  Process Biochemistry. Volume 50, Issue 3, March  2015, Pages 347-356  http://dx.doi.org/10.1016/j.procbio.2014.11.014 GPL14656 | | Microarray | GSE55516 | GSM1338519 | wt x Furfural(13mM) |
| Park et. al. | 2015 | Park, H.-S., Um, Y., Sim, S.J., Lee, S.Y., *Woo,  H.M., Transcriptomic analysis of  Corynebacterium glutamicum to overcome the toxicity of furfural present in hydrolysates.  Process Biochemistry. Volume 50, Issue 3, March  2015, Pages 347-356  http://dx.doi.org/10.1016/j.procbio.2014.11.014 GPL14656 | | Microarray | GSE55516 | GSM1338520 | wt x Furfural(13mM) |
| Park et. al. | 2015 | Park, H.-S., Um, Y., Sim, S.J., Lee, S.Y., *Woo,  H.M., Transcriptomic analysis of  Corynebacterium glutamicum to overcome the toxicity of furfural present in hydrolysates.  Process Biochemistry. Volume 50, Issue 3, March  2015, Pages 347-356  http://dx.doi.org/10.1016/j.procbio.2014.11.014 GPL14656 | | Microarray | GSE55516 | GSM1338521 | wt x Furfural(20mM) |

21;19(1):11. PMID: 31964372 GPL9860 GSE140735 GSM4182914 AmyE_noSP_induced x wt Jurischka et. al. 2020

Jurischka S, Bida A, Dohmen-Olma D, Kleine B et al. A secretion biosensor for monitoring Secdependent protein export in Corynebacterium glutamicum. Microb Cell Fact 2020 Jan

21;19(1):11. PMID: 31964372 GPL9860 GSE140735 GSM4182915 wt x AmyE_noSP_induced

Park et. al. 2015

Park, H.-S., Um, Y., Sim, S.J., Lee, S.Y., *Woo,

H.M., Transcriptomic analysis of

Corynebacterium glutamicum to overcome the toxicity of furfural present in hydrolysates.

Process Biochemistry. Volume 50, Issue 3, March

2015, Pages 347-356

| Lee et. al. | 2016 | Lee J, Saddler JN, Um Y, Woo HM. Adaptive evolution and metabolic engineering of a cellobiose- and xylose- negative Corynebacterium glutamicum that co-utilizes cellobiose and xylose.  Microb Cell Fact 2016 Jan 22;15:20. PMID:  26801253 GPL14656 | |  | GSE65076 | GSM1586873 | Cg-Cello01(evo)-glu-1 |
| --- | --- | --- | --- | --- | --- | --- | --- |
| Lee et. al. | 2016 | Lee J, Saddler JN, Um Y, Woo HM. Adaptive evolution and metabolic engineering of a cellobiose- and xylose- negative Corynebacterium glutamicum that co-utilizes cellobiose and xylose.  Microb Cell Fact 2016 Jan 22;15:20. PMID:  26801253 GPL14656 | | Microarray | GSE65076 | GSM1586874 | Cg-Cello01(evo)-glu-2 |
| Lee et. al. | 2016 | Lee J, Saddler JN, Um Y, Woo HM. Adaptive evolution and metabolic engineering of a cellobiose- and xylose- negative Corynebacterium glutamicum that co-utilizes cellobiose and xylose.  Microb Cell Fact 2016 Jan 22;15:20. PMID:  26801253 GPL14656 | | Microarray | GSE65076 | GSM1586875 | Cg-Cello01(evo)-cello-2 |
| Lee et. al. | 2016 | Lee J, Saddler JN, Um Y, Woo HM. Adaptive evolution and metabolic engineering of a cellobiose- and xylose- negative Corynebacterium glutamicum that co-utilizes cellobiose and xylose.  Microb Cell Fact 2016 Jan 22;15:20. PMID:  26801253 GPL14656 | | Microarray | GSE65076 | GSM1586876 | Cg-Cello02(evo)-glu-1 |
| Lee et. al. | 2016 | Lee J, Saddler JN, Um Y, Woo HM. Adaptive evolution and metabolic engineering of a cellobiose- and xylose- negative Corynebacterium glutamicum that co-utilizes cellobiose and xylose.  Microb Cell Fact 2016 Jan 22;15:20. PMID:  26801253 GPL14656 | | Microarray | GSE65076 | GSM1586877 | Cg-Cello02(evo)-glu-2 |
| Lee et. al. | 2016 | Lee J, Saddler JN, Um Y, Woo HM. Adaptive evolution and metabolic engineering of a cellobiose- and xylose- negative Corynebacterium glutamicum that co-utilizes cellobiose and xylose. | | Microarray | GSE65076 | GSM1586878 | Cg-Cello02(evo)-cello-2 |
|  |  | Microb Cell Fact 2016 Jan 22;15:20. PMID:  26801253 | GPL14656 |  |  |  |  |
| Park et. al. | 2013 | Park SH, Kim HU, Kim TY, Park JS et al. Metabolic engineering of Corynebacterium glutamicum for  L-arginine production. Nat Commun 2014 Aug  5;5:4618. PMID: 25091334 | GPL14656 | Microarray | GSE52737 | GSM1275140 | AR2 |
| Park et. al. | 2013 | Park SH, Kim HU, Kim TY, Park JS et al. Metabolic engineering of Corynebacterium glutamicum for  L-arginine production. Nat Commun 2014 Aug  5;5:4618. PMID: 25091334 | GPL14656 | Microarray | GSE52737 | GSM1275141 | AR2 |
| Park et. al. | 2013 | Park SH, Kim HU, Kim TY, Park JS et al. Metabolic engineering of Corynebacterium glutamicum for  L-arginine production. Nat Commun 2014 Aug  5;5:4618. PMID: 25091334 | GPL14656 | Microarray | GSE52737 | GSM1275142 | AR6 |
| Park et. al. | 2013 | Park SH, Kim HU, Kim TY, Park JS et al. Metabolic engineering of Corynebacterium glutamicum for  L-arginine production. Nat Commun 2014 Aug  5;5:4618. PMID: 25091334 | GPL14656 | Microarray | GSE52737 | GSM1275143 | AR6 |
| Heyer et. al. | 2012 | Heyer A, Gätgens C, Hentschel E, Kalinowski J et al. The two-component system ChrSA is crucial for haem tolerance and interferes with HrrSA in  haem-dependent gene regulation in |  | Microarray | GSE37327 | GSM916158 | WT_Fe x Delta-chrSA_Fe |
|  |  | Corynebacterium glutamicum. Microbiology 2012  Dec;158(Pt 12):3020-3031. PMID: 23038807 GPL15451 | |  |  |  |  |
| Heyer et. al. | 2012 | Heyer A, Gätgens C, Hentschel E, Kalinowski J et al. The two-component system ChrSA is crucial for haem tolerance and interferes with HrrSA in  haem-dependent gene regulation in  Corynebacterium glutamicum. Microbiology 2012  Dec;158(Pt 12):3020-3031. PMID: 23038807 GPL15451 | | Microarray | GSE37327 | GSM916159 | WT_Fe x Delta-chrSA_Fe |
| Heyer et. al. | 2012 | Heyer A, Gätgens C, Hentschel E, Kalinowski J et al. The two-component system ChrSA is crucial for haem tolerance and interferes with HrrSA in  haem-dependent gene regulation in  Corynebacterium glutamicum. Microbiology 2012  Dec;158(Pt 12):3020-3031. PMID: 23038807 GPL15451 | | Microarray | GSE37327 | GSM916160 | Delta-chrSA_Fe x WT_Fe |
| Heyer et. al. | 2012 | Heyer A, Gätgens C, Hentschel E, Kalinowski J et al. The two-component system ChrSA is crucial for haem tolerance and interferes with HrrSA in  haem-dependent gene regulation in  Corynebacterium glutamicum. Microbiology 2012  Dec;158(Pt 12):3020-3031. PMID: 23038807 GPL15451 | | Microarray | GSE37327 | GSM916161 | WT_Heme x Delta-chrSA_Heme |

http://dx.doi.org/10.1016/j.procbio.2014.11.014 GPL14656 GSE55516 GSM1338522 wt x Furfural(20mM) Lee et. al. 2016

Lee J, Saddler JN, Um Y, Woo HM. Adaptive evolution and metabolic engineering of a cellobiose- and xylose- negative Corynebacterium glutamicum that co-utilizes cellobiose and xylose.

Microb Cell Fact 2016 Jan 22;15:20. PMID:

26801253 GPL14656 GSE65076 GSM1586872 Wt

Heyer et. al. 2012

Heyer A, Gätgens C, Hentschel E, Kalinowski J et al. The two-component system ChrSA is crucial for haem tolerance and interferes with HrrSA in haem-dependent gene regulation in

Corynebacterium glutamicum. Microbiology 2012

Dec;158(Pt 12):3020-3031. PMID: 23038807 GPL15451 GSE37327 GSM916162 WT_Heme x Delta-chrSA_Heme

| Klaffl et. al. | 2013 | Klaffl S, Brocker M, Kalinowski J, Eikmanns BJ et al. Complex regulation of the  phosphoenolpyruvate carboxykinase gene pck and characterization of its GntR-type regulator IolR as a repressor of myo-inositol utilization genes in Corynebacterium glutamicum. J  Bacteriol 2013 Sep;195(18):4283-96. PMID:  23873914 | GPL15451 | Microarray | GSE44812 | GSM1091989 | WT x Delta-iolR |
| --- | --- | --- | --- | --- | --- | --- | --- |
| Klaffl et. al. | 2013 | Klaffl S, Brocker M, Kalinowski J, Eikmanns BJ et al. Complex regulation of the  phosphoenolpyruvate carboxykinase gene pck and characterization of its GntR-type regulator IolR as a repressor of myo-inositol utilization genes in Corynebacterium glutamicum. J  Bacteriol 2013 Sep;195(18):4283-96. PMID:  23873914 | GPL15451 | Microarray | GSE44812 | GSM1091990 | Delta-iolR x WT |
| Klaffl et. al. | 2013 | Klaffl S, Brocker M, Kalinowski J, Eikmanns BJ et al. Complex regulation of the  phosphoenolpyruvate carboxykinase gene pck and characterization of its GntR-type regulator IolR as a repressor of myo-inositol utilization genes in Corynebacterium glutamicum. J  Bacteriol 2013 Sep;195(18):4283-96. PMID:  23873914 | GPL15451 | Microarray | GSE44812 | GSM1091991 | WT x Delta-iolR |
| Witthoff et. al. | 2013 | Witthoff S, Mühlroth A, Marienhagen J, Bott M. C1 metabolism in Corynebacterium glutamicum:  an endogenous pathway for oxidation of methanol to carbon dioxide. Appl Environ Microbiol 2013 Nov;79(22):6974-83. PMID:  24014532 | GPL15451 | Microarray | GSE49936 | GSM1210286 | CgWT_Glu x CgWT_Glu_MeOH |
| Witthoff et. al. | 2013 | Witthoff S, Mühlroth A, Marienhagen J, Bott M. C1 metabolism in Corynebacterium glutamicum:  an endogenous pathway for oxidation of methanol to carbon dioxide. Appl Environ Microbiol 2013 Nov;79(22):6974-83. PMID:  24014532 | GPL15451 | Microarray | GSE49936 | GSM1210288 | CgWT_Glu x CgWT_Glu_MeOH |
| Witthoff et. al. | 2013 | Witthoff S, Mühlroth A, Marienhagen J, Bott M. C1 metabolism in Corynebacterium glutamicum:  an endogenous pathway for oxidation of methanol to carbon dioxide. Appl Environ Microbiol 2013 Nov;79(22):6974-83. PMID:  24014532 | GPL15451 | Microarray | GSE49936 | GSM1210289 | CgWT_noC x CgWT_noC_MeOH |
| Witthoff et. al. | 2013 | Witthoff S, Mühlroth A, Marienhagen J, Bott M. C1 metabolism in Corynebacterium glutamicum:  an endogenous pathway for oxidation of methanol to carbon dioxide. Appl Environ Microbiol 2013 Nov;79(22):6974-83. PMID:  24014532 | GPL15451 | Microarray | GSE49936 | GSM1210290 | CgWT_noC_MeOH x CgWT_noC |
| Kallscheuer et. al. | 2015 | Kallscheuer N, Bott M, van Ooyen J, Polen T. |  | Microarray | GSE65294 | GSM1591777 | JVO1_DfkpA x JVO1 |
|  |  | Single-Domain Peptidyl-Prolyl cis/trans Isomerase  FkpA from Corynebacterium glutamicum  Improves the Biomass Yield at Increased Growth  Temperatures. Appl Environ Microbiol 2015  Nov;81(22):7839-50. PMID: 26341203 GPL15451 | |  |  |  |  |
| Kallscheuer et. al. | 2015 | Kallscheuer N, Bott M, van Ooyen J, Polen T.  Single-Domain Peptidyl-Prolyl cis/trans Isomerase  FkpA from Corynebacterium glutamicum  Improves the Biomass Yield at Increased Growth  Temperatures. Appl Environ Microbiol 2015  Nov;81(22):7839-50. PMID: 26341203 GPL15451 | | Microarray | GSE65294 | GSM1591778 | JVO1_DfkpA x JVO1 |
| Morosov et. al. | 2018 | Morosov X, Davoudi CF, Baumgart M, Brocker M et al. The copper-deprivation stimulon of <i>Corynebacterium glutamicum</i> comprises proteins for biogenesis of the actinobacterial cytochrome <i>bc</i> <sub>1</sub>-<i>aa</i>  <sub>3</sub> supercomplex. J Biol Chem 2018  Oct 5;293(40):15628-15640. PMID: 30154248 GPL15451 | | Microarray | GSE117530 | GSM3302526 | copper sufficiency x copper starvation |
| Morosov et. al. | 2018 | Morosov X, Davoudi CF, Baumgart M, Brocker M et al. The copper-deprivation stimulon of <i>Corynebacterium glutamicum</i> comprises proteins for biogenesis of the actinobacterial cytochrome <i>bc</i> <sub>1</sub>-<i>aa</i>  <sub>3</sub> supercomplex. J Biol Chem 2018  Oct 5;293(40):15628-15640. PMID: 30154248 GPL15451 | | Microarray | GSE117530 | GSM3302527 | copper sufficiency x copper starvation |
| Morosov et. al. | 2018 | Morosov X, Davoudi CF, Baumgart M, Brocker M et al. The copper-deprivation stimulon of <i>Corynebacterium glutamicum</i> comprises proteins for biogenesis of the actinobacterial cytochrome <i>bc</i> <sub>1</sub>-<i>aa</i>  <sub>3</sub> supercomplex. J Biol Chem 2018  Oct 5;293(40):15628-15640. PMID: 30154248 GPL15451 | | Microarray | GSE117530 | GSM3302528 | copper starvation x copper sufficiency |

Heyer et. al. 2012

Heyer A, Gätgens C, Hentschel E, Kalinowski J et al. The two-component system ChrSA is crucial for haem tolerance and interferes with HrrSA in haem-dependent gene regulation in

Corynebacterium glutamicum. Microbiology 2012

Dec;158(Pt 12):3020-3031. PMID: 23038807 GPL15451 Microarray GSE37327 GSM916163 Delta-chrSA_Heme x WT_Heme

Donovan et. al. 2015

Donovan C, Heyer A, Pfeifer E, Polen T et al. A prophage-encoded actin-like protein required for efficient viral DNA replication in bacteria. Nucleic Acids Res 2015 May 26;43(10):5002-16. PMID:

| Donovan et. al. | 2015 | Donovan C, Heyer A, Pfeifer E, Polen T et al. A prophage-encoded actin-like protein required for efficient viral DNA replication in bacteria. Nucleic Acids Res 2015 May 26;43(10):5002-16. PMID:  25916847 GPL16988 | | Microarray | GSE45905 | GSM1119373 | WT_without_MmC_6h x WT_with_MmC_6h |
| --- | --- | --- | --- | --- | --- | --- | --- |
| Donovan et. al. | 2015 | Donovan C, Heyer A, Pfeifer E, Polen T et al. A prophage-encoded actin-like protein required for efficient viral DNA replication in bacteria. Nucleic Acids Res 2015 May 26;43(10):5002-16. PMID:  25916847 GPL16988 | | Microarray | GSE45906 | GSM1119374 | WT_without_MmC_1h x WT_with_MmC_1h |
| Donovan et. al. | 2015 | Donovan C, Heyer A, Pfeifer E, Polen T et al. A prophage-encoded actin-like protein required for efficient viral DNA replication in bacteria. Nucleic Acids Res 2015 May 26;43(10):5002-16. PMID:  25916847 GPL16988 | | Microarray | GSE45906 | GSM1119375 | WT_with_MmC_1h x WT_without_MmC_1h |
| Donovan et. al. | 2015 | Donovan C, Heyer A, Pfeifer E, Polen T et al. A prophage-encoded actin-like protein required for efficient viral DNA replication in bacteria. Nucleic Acids Res 2015 May 26;43(10):5002-16. PMID:  25916847 GPL16988 | | Microarray | GSE45906 | GSM1119376 | WT_without_MmC_3h x WT_with_MmC_3h |
| Donovan et. al. | 2015 | Donovan C, Heyer A, Pfeifer E, Polen T et al. A prophage-encoded actin-like protein required for efficient viral DNA replication in bacteria. Nucleic Acids Res 2015 May 26;43(10):5002-16. PMID:  25916847 GPL16988 | | Microarray | GSE45906 | GSM1119377 | WT_with_MmC_3h x WT_without_MmC_3h |
| Donovan et. al. | 2015 | Donovan C, Heyer A, Pfeifer E, Polen T et al. A prophage-encoded actin-like protein required for efficient viral DNA replication in bacteria. Nucleic Acids Res 2015 May 26;43(10):5002-16. PMID:  25916847 GPL16988 | | Microarray | GSE45906 | GSM1119378 | WT_without_MmC_6h x WT_with_MmC_6h |
| Donovan et. al. | 2015 | Donovan C, Heyer A, Pfeifer E, Polen T et al. A prophage-encoded actin-like protein required for efficient viral DNA replication in bacteria. Nucleic | | Microarray | GSE45906 | GSM1119379 | WT_with_MmC_6h x WT_without_MmC_6h |
|  |  | Acids Res 2015 May 26;43(10):5002-16. PMID:  25916847 | GPL16988 |  |  |  |  |
| Küberl et. al. | 2014 | Küberl A, Fränzel B, Eggeling L, Polen T et al. Pupylated proteins in Corynebacterium glutamicum revealed by MudPIT analysis.  Proteomics 2014 Jun;14(12):1531-42. PMID:  24737727 | GPL16988 | Microarray | GSE48038 | GSM1166145 | WT x delta_pup |
| Küberl et. al. | 2014 | Küberl A, Fränzel B, Eggeling L, Polen T et al. Pupylated proteins in Corynebacterium glutamicum revealed by MudPIT analysis.  Proteomics 2014 Jun;14(12):1531-42. PMID:  24737727 | GPL16988 | Microarray | GSE48038 | GSM1166146 | WT x delta_pup |
| Küberl et. al. | 2014 | Küberl A, Fränzel B, Eggeling L, Polen T et al. Pupylated proteins in Corynebacterium glutamicum revealed by MudPIT analysis.  Proteomics 2014 Jun;14(12):1531-42. PMID:  24737727 | GPL16988 | Microarray | GSE48038 | GSM1166147 | WT x delta_pup |
| Küberl et. al. | 2014 | Küberl A, Fränzel B, Eggeling L, Polen T et al. Pupylated proteins in Corynebacterium glutamicum revealed by MudPIT analysis.  Proteomics 2014 Jun;14(12):1531-42. PMID:  24737727 | GPL16988 | Microarray | GSE48038 | GSM1166148 | BHI_WT x BHI_delta_pup |
| Küberl et. al. | 2014 | Küberl A, Fränzel B, Eggeling L, Polen T et al. Pupylated proteins in Corynebacterium glutamicum revealed by MudPIT analysis.  Proteomics 2014 Jun;14(12):1531-42. PMID:  24737727 | GPL16988 | Microarray | GSE48038 | GSM1166149 | BHI_delta_pup x BHI_WT |
| Küberl et. al. | 2014 | Küberl A, Fränzel B, Eggeling L, Polen T et al. Pupylated proteins in Corynebacterium glutamicum revealed by MudPIT analysis.  Proteomics 2014 Jun;14(12):1531-42. PMID:  24737727 | GPL16988 | Microarray | GSE48038 | GSM1166150 | BHI_WT x BHI_delta_pup |
| Baumgart et. al. | 2014 | Baumgart M, Luder K, Grover S, Gätgens C et al.  IpsA, a novel LacI-type regulator, is required for |  | Microarray | GSE50210 | GSM1215809 | wt x Δcg2910 |
|  |  | inositol-derived lipid formation in Corynebacteria and Mycobacteria. BMC Biol 2013 Dec 30;11:122.  PMID: 24377418 GPL16988 | |  |  |  |  |
| Baumgart et. al. | 2014 | Baumgart M, Luder K, Grover S, Gätgens C et al. IpsA, a novel LacI-type regulator, is required for inositol-derived lipid formation in Corynebacteria and Mycobacteria. BMC Biol 2013 Dec 30;11:122.  PMID: 24377418 GPL16988 | | Microarray | GSE50210 | GSM1215810 | wt x Δcg2910 |
| Baumgart et. al. | 2014 | Baumgart M, Luder K, Grover S, Gätgens C et al. IpsA, a novel LacI-type regulator, is required for inositol-derived lipid formation in Corynebacteria and Mycobacteria. BMC Biol 2013 Dec 30;11:122.  PMID: 24377418 GPL16988 | | Microarray | GSE50210 | GSM1215811 | Δcg2910 x wt |
| Donovan et. al. | 2015 | Donovan C, Heyer A, Pfeifer E, Polen T et al. A prophage-encoded actin-like protein required for efficient viral DNA replication in bacteria. Nucleic Acids Res 2015 May 26;43(10):5002-16. PMID:  25916847 GPL16989 | | Microarray | GSE45906 | GSM1119374 | WT_without_MmC_1h x WT_with_MmC_1h |
| Donovan et. al. | 2015 | Donovan C, Heyer A, Pfeifer E, Polen T et al. A prophage-encoded actin-like protein required for efficient viral DNA replication in bacteria. Nucleic Acids Res 2015 May 26;43(10):5002-16. PMID:  25916847 GPL16989 | | Microarray | GSE45906 | GSM1119375 | WT_with_MmC_1h x WT_without_MmC_1h |

25916847 GPL16988 GSE45905 GSM1119371 WT_without_MmC_1h x WT_with_MmC_1h Donovan et. al. 2015

Donovan C, Heyer A, Pfeifer E, Polen T et al. A prophage-encoded actin-like protein required for efficient viral DNA replication in bacteria. Nucleic Acids Res 2015 May 26;43(10):5002-16. PMID:

25916847 GPL16988 Microarray GSE45905 GSM1119372 WT_without_MmC_3h x WT_with_MmC_3h

Donovan et. al. 2015

Donovan C, Heyer A, Pfeifer E, Polen T et al. A prophage-encoded actin-like protein required for efficient viral DNA replication in bacteria. Nucleic Acids Res 2015 May 26;43(10):5002-16. PMID:

| Donovan et. al. | 2015 | Donovan C, Heyer A, Pfeifer E, Polen T et al. A prophage-encoded actin-like protein required for efficient viral DNA replication in bacteria. Nucleic Acids Res 2015 May 26;43(10):5002-16. PMID:  25916847 GPL16989 | | Microarray | GSE45906 | GSM1119378 | WT_without_MmC_6h x WT_with_MmC_6h |
| --- | --- | --- | --- | --- | --- | --- | --- |
| Donovan et. al. | 2015 | Donovan C, Heyer A, Pfeifer E, Polen T et al. A prophage-encoded actin-like protein required for efficient viral DNA replication in bacteria. Nucleic Acids Res 2015 May 26;43(10):5002-16. PMID:  25916847 GPL16989 | | Microarray | GSE45906 | GSM1119379 | WT_with_MmC_6h x WT_without_MmC_6h |
| Donovan et. al. | 2015 | Donovan C, Heyer A, Pfeifer E, Polen T et al. A prophage-encoded actin-like protein required for efficient viral DNA replication in bacteria. Nucleic Acids Res 2015 May 26;43(10):5002-16. PMID:  25916847 GPL16989 | | Microarray | GSE45905 | GSM1119371 | WT_without_MmC_1h x WT_with_MmC_1h |
| Donovan et. al. | 2015 | Donovan C, Heyer A, Pfeifer E, Polen T et al. A prophage-encoded actin-like protein required for efficient viral DNA replication in bacteria. Nucleic Acids Res 2015 May 26;43(10):5002-16. PMID:  25916847 GPL16989 | | Microarray | GSE45905 | GSM1119372 | WT_without_MmC_3h x WT_with_MmC_3h |
| Donovan et. al. | 2015 | Donovan C, Heyer A, Pfeifer E, Polen T et al. A prophage-encoded actin-like protein required for efficient viral DNA replication in bacteria. Nucleic | | Microarray | GSE45905 | GSM1119373 | WT_without_MmC_6h x WT_with_MmC_6h |
|  |  | Acids Res 2015 May 26;43(10):5002-16. PMID:  25916847 | GPL16989 |  |  |  |  |
| Küberl et. al. | 2014 | Küberl A, Fränzel B, Eggeling L, Polen T et al. Pupylated proteins in Corynebacterium glutamicum revealed by MudPIT analysis.  Proteomics 2014 Jun;14(12):1531-42. PMID:  24737727 | GPL16989 | Microarray | GSE48038 | GSM1166145 | WT x delta_pup |
| Küberl et. al. | 2014 | Küberl A, Fränzel B, Eggeling L, Polen T et al. Pupylated proteins in Corynebacterium glutamicum revealed by MudPIT analysis.  Proteomics 2014 Jun;14(12):1531-42. PMID:  24737727 | GPL16989 | Microarray | GSE48038 | GSM1166146 | WT x delta_pup |
| Küberl et. al. | 2014 | Küberl A, Fränzel B, Eggeling L, Polen T et al. Pupylated proteins in Corynebacterium glutamicum revealed by MudPIT analysis.  Proteomics 2014 Jun;14(12):1531-42. PMID:  24737727 | GPL16989 | Microarray | GSE48038 | GSM1166147 | WT x delta_pup |
| Küberl et. al. | 2014 | Küberl A, Fränzel B, Eggeling L, Polen T et al. Pupylated proteins in Corynebacterium glutamicum revealed by MudPIT analysis.  Proteomics 2014 Jun;14(12):1531-42. PMID:  24737727 | GPL16989 | Microarray | GSE48038 | GSM1166148 | BHI_WT x BHI_delta_pup |
| Küberl et. al. | 2014 | Küberl A, Fränzel B, Eggeling L, Polen T et al. Pupylated proteins in Corynebacterium glutamicum revealed by MudPIT analysis.  Proteomics 2014 Jun;14(12):1531-42. PMID:  24737727 | GPL16989 | Microarray | GSE48038 | GSM1166149 | BHI_delta_pup x BHI_WT |
| Küberl et. al. | 2014 | Küberl A, Fränzel B, Eggeling L, Polen T et al. Pupylated proteins in Corynebacterium glutamicum revealed by MudPIT analysis.  Proteomics 2014 Jun;14(12):1531-42. PMID:  24737727 | GPL16989 | Microarray | GSE48038 | GSM1166150 | BHI_WT x BHI_delta_pup |
| Küberl et. al. | 2016 | Küberl A, Polen T, Bott M. The pupylation machinery is involved in iron homeostasis by targeting the iron storage protein ferritin. Proc |  | Microarray | GSE64866 | GSM1581851 | wild_type x delta_pup |
|  |  | Natl Acad Sci U S A 2016 Apr 26;113(17):4806-11.  PMID: 27078093 GPL16989 | |  |  |  |  |
| Küberl et. al. | 2016 | Küberl A, Polen T, Bott M. The pupylation machinery is involved in iron homeostasis by targeting the iron storage protein ferritin. Proc  Natl Acad Sci U S A 2016 Apr 26;113(17):4806-11.  PMID: 27078093 GPL16989 | | Microarray | GSE64866 | GSM1581852 | wild_type x delta_pup |
| Küberl et. al. | 2016 | Küberl A, Polen T, Bott M. The pupylation machinery is involved in iron homeostasis by targeting the iron storage protein ferritin. Proc  Natl Acad Sci U S A 2016 Apr 26;113(17):4806-11.  PMID: 27078093 GPL16989 | | Microarray | GSE64866 | GSM1581853 | delta_pup x wild_type |
| Kallscheuer et. al. | 2015 | Kallscheuer N, Vogt M, Kappelmann J, Krumbach K et al. Identification of the phd gene cluster responsible for phenylpropanoid utilization in Corynebacterium glutamicum. Appl Microbiol Biotechnol 2016 Feb;100(4):1871-1881. PMID:  26610800 GPL16989 | | Microarray | GSE69412 | GSM1700868 | control x pulse |
| Kallscheuer et. al. | 2015 | Kallscheuer N, Vogt M, Kappelmann J, Krumbach K et al. Identification of the phd gene cluster responsible for phenylpropanoid utilization in Corynebacterium glutamicum. Appl Microbiol Biotechnol 2016 Feb;100(4):1871-1881. PMID:  26610800 GPL16989 | | Microarray | GSE69412 | GSM1700869 | control x pulse |
| Kallscheuer et. al. | 2015 | Kallscheuer N, Vogt M, Kappelmann J, Krumbach K et al. Identification of the phd gene cluster responsible for phenylpropanoid utilization in Corynebacterium glutamicum. Appl Microbiol Biotechnol 2016 Feb;100(4):1871-1881. PMID:  26610800 GPL16989 | | Microarray | GSE69413 | GSM1700870 | glutamicum_control x pulse |

25916847 GPL16989 Microarray GSE45906 GSM1119376 WT_without_MmC_3h x WT_with_MmC_3h Donovan et. al. 2015

Donovan C, Heyer A, Pfeifer E, Polen T et al. A prophage-encoded actin-like protein required for efficient viral DNA replication in bacteria. Nucleic Acids Res 2015 May 26;43(10):5002-16. PMID:

25916847 GPL16989 Microarray GSE45906 GSM1119377 WT_with_MmC_3h x WT_without_MmC_3h

Kallscheuer et. al. 2015

Kallscheuer N, Vogt M, Kappelmann J, Krumbach K et al. Identification of the phd gene cluster responsible for phenylpropanoid utilization in Corynebacterium glutamicum. Appl Microbiol Biotechnol 2016 Feb;100(4):1871-1881. PMID:

| Kallscheuer et. al. | 2015 | Kallscheuer N, Vogt M, Kappelmann J, Krumbach K et al. Identification of the phd gene cluster responsible for phenylpropanoid utilization in Corynebacterium glutamicum. Appl Microbiol Biotechnol 2016 Feb;100(4):1871-1881. PMID:  26610800 | GPL16989 | Microarray | GSE69413 | GSM1700873 | glutamicum_control x pulse |
| --- | --- | --- | --- | --- | --- | --- | --- |
| Kallscheuer et. al. | 2015 | Kallscheuer N, Vogt M, Kappelmann J, Krumbach K et al. Identification of the phd gene cluster responsible for phenylpropanoid utilization in Corynebacterium glutamicum. Appl Microbiol Biotechnol 2016 Feb;100(4):1871-1881. PMID:  26610800 | GPL16989 | Microarray | GSE69413 | GSM1700874 | glutamicum_control x Δcg0343 |
| Kallscheuer et. al. | 2015 | Kallscheuer N, Vogt M, Kappelmann J, Krumbach K et al. Identification of the phd gene cluster responsible for phenylpropanoid utilization in Corynebacterium glutamicum. Appl Microbiol Biotechnol 2016 Feb;100(4):1871-1881. PMID:  26610800 | GPL16989 | Microarray | GSE69413 | GSM1700875 | glutamicum_control x Δcg0343 |
| Kallscheuer et. al. | 2015 | Kallscheuer N, Vogt M, Kappelmann J, Krumbach K et al. Identification of the phd gene cluster responsible for phenylpropanoid utilization in Corynebacterium glutamicum. Appl Microbiol Biotechnol 2016 Feb;100(4):1871-1881. PMID:  26610800 | GPL16989 | Microarray | GSE69413 | GSM1700876 | glutamicum_control x pulse |
| Kallscheuer et. al. | 2015 | Kallscheuer N, Vogt M, Kappelmann J, Krumbach K et al. Identification of the phd gene cluster responsible for phenylpropanoid utilization in Corynebacterium glutamicum. Appl Microbiol Biotechnol 2016 Feb;100(4):1871-1881. PMID:  26610800 | GPL16989 | Microarray | GSE69413 | GSM1700877 | glutamicum_control x pulse |
| Kallscheuer et. al. | 2015 | Kallscheuer N, Vogt M, Kappelmann J, Krumbach K et al. Identification of the phd gene cluster responsible for phenylpropanoid utilization in Corynebacterium glutamicum. Appl Microbiol Biotechnol 2016 Feb;100(4):1871-1881. PMID:  26610800 | GPL16989 | Microarray | GSE69413 | GSM1700878 | glutamicum_control x pulse |
| Kallscheuer et. al. | 2015 | Kallscheuer N, Vogt M, Kappelmann J, Krumbach K et al. Identification of the phd gene cluster responsible for phenylpropanoid utilization in Corynebacterium glutamicum. Appl Microbiol Biotechnol 2016 Feb;100(4):1871-1881. PMID:  26610800 | GPL16989 | Microarray | GSE69413 | GSM1700879 | glutamicum_control x pulse |
| Kallscheuer et. al. | 2015 | Kallscheuer N, Vogt M, Kappelmann J, Krumbach K et al. Identification of the phd gene cluster responsible for phenylpropanoid utilization in Corynebacterium glutamicum. Appl Microbiol Biotechnol 2016 Feb;100(4):1871-1881. PMID:  26610800 | GPL16989 | Microarray | GSE69413 | GSM1700880 | glutamicum_control x pulse |
| Kallscheuer et. al. | 2015 | Kallscheuer N, Vogt M, Kappelmann J, Krumbach K et al. Identification of the phd gene cluster responsible for phenylpropanoid utilization in Corynebacterium glutamicum. Appl Microbiol Biotechnol 2016 Feb;100(4):1871-1881. PMID:  26610800 | GPL16989 | Microarray | GSE69413 | GSM1700881 | glutamicum_control x pulse |
| Kallscheuer et. al. | 2015 | Kallscheuer N, Vogt M, Kappelmann J, Krumbach K et al. Identification of the phd gene cluster responsible for phenylpropanoid utilization in Corynebacterium glutamicum. Appl Microbiol Biotechnol 2016 Feb;100(4):1871-1881. PMID:  26610800 | GPL16989 | Microarray | GSE69413 | GSM1700882 | glutamicum_control x pulse |
| Kallscheuer et. al. | 2015 | Kallscheuer N, Vogt M, Kappelmann J, Krumbach K et al. Identification of the phd gene cluster responsible for phenylpropanoid utilization in Corynebacterium glutamicum. Appl Microbiol Biotechnol 2016 Feb;100(4):1871-1881. PMID:  26610800 | GPL16989 | Microarray | GSE69413 | GSM1700883 | glutamicum_control x pulse |
| Kraxner et. al. | 2019 | Kraxner KJ, Polen T, Baumgart M, Bott M. The conserved actinobacterial transcriptional regulator FtsR controls expression of ftsZ and further target genes and influences growth and cell division in Corynebacterium glutamicum.  BMC Microbiol 2019 Aug 5;19(1):179. PMID:  31382874 | GPL16989 | Microarray | GSE107921 | GSM2883410 | glucose x Δcg1631 |

26610800 GPL16989 Microarray GSE69413 GSM1700871 glutamicum_control x pulse Kallscheuer et. al. 2015

Kallscheuer N, Vogt M, Kappelmann J, Krumbach K et al. Identification of the phd gene cluster responsible for phenylpropanoid utilization in Corynebacterium glutamicum. Appl Microbiol Biotechnol 2016 Feb;100(4):1871-1881. PMID:

26610800 GPL16989 Microarray GSE69413 GSM1700872 glutamicum_control x pulse

Kraxner et. al. 2019

Kraxner KJ, Polen T, Baumgart M, Bott M. The conserved actinobacterial transcriptional regulator FtsR controls expression of ftsZ and further target genes and influences growth and cell division in Corynebacterium glutamicum.

BMC Microbiol 2019 Aug 5;19(1):179. PMID:

| Morosov et. al. | 2018 | Morosov X, Davoudi CF, Baumgart M, Brocker M et al. The copper-deprivation stimulon of <i>Corynebacterium glutamicum</i> comprises proteins for biogenesis of the actinobacterial cytochrome <i>bc</i> <sub>1</sub>-<i>aa</i> <sub>3</sub> supercomplex. J Biol Chem 2018  Oct 5;293(40):15628-15640. PMID: 30154248 | GPL16989 | Microarray | GSE117566 | GSM3303967 | wt x Δcg2699 |
| --- | --- | --- | --- | --- | --- | --- | --- |
| Morosov et. al. | 2018 | Morosov X, Davoudi CF, Baumgart M, Brocker M et al. The copper-deprivation stimulon of <i>Corynebacterium glutamicum</i> comprises proteins for biogenesis of the actinobacterial cytochrome <i>bc</i> <sub>1</sub>-<i>aa</i> <sub>3</sub> supercomplex. J Biol Chem 2018  Oct 5;293(40):15628-15640. PMID: 30154248 | GPL16989 | Microarray | GSE117566 | GSM3303968 | wt x Δcg2699 |
| Morosov et. al. | 2018 | Morosov X, Davoudi CF, Baumgart M, Brocker M et al. The copper-deprivation stimulon of <i>Corynebacterium glutamicum</i> comprises proteins for biogenesis of the actinobacterial cytochrome <i>bc</i> <sub>1</sub>-<i>aa</i> <sub>3</sub> supercomplex. J Biol Chem 2018  Oct 5;293(40):15628-15640. PMID: 30154248 | GPL16989 | Microarray | GSE117566 | GSM3303969 | Δcg2699 x wt |
| Küberl et. al. | 2014 | Küberl A, Fränzel B, Eggeling L, Polen T et al. Pupylated proteins in Corynebacterium glutamicum revealed by MudPIT analysis.  Proteomics 2014 Jun;14(12):1531-42. PMID:  24737727 | GPL17304 | Microarray | GSE48038 | GSM1166145 | WT x delta_pup |
| Küberl et. al. | 2014 | Küberl A, Fränzel B, Eggeling L, Polen T et al. Pupylated proteins in Corynebacterium glutamicum revealed by MudPIT analysis.  Proteomics 2014 Jun;14(12):1531-42. PMID:  24737727 | GPL17304 | Microarray | GSE48038 | GSM1166146 | WT x delta_pup |
| Küberl et. al. | 2014 | Küberl A, Fränzel B, Eggeling L, Polen T et al. Pupylated proteins in Corynebacterium glutamicum revealed by MudPIT analysis.  Proteomics 2014 Jun;14(12):1531-42. PMID:  24737727 | GPL17304 | Microarray | GSE48038 | GSM1166147 | WT x delta_pup |
| Küberl et. al. | 2014 | Küberl A, Fränzel B, Eggeling L, Polen T et al. Pupylated proteins in Corynebacterium glutamicum revealed by MudPIT analysis.  Proteomics 2014 Jun;14(12):1531-42. PMID:  24737727 | GPL17304 | Microarray | GSE48038 | GSM1166148 | BHI_WT x BHI_delta_pup |
| Küberl et. al. | 2014 | Küberl A, Fränzel B, Eggeling L, Polen T et al. Pupylated proteins in Corynebacterium glutamicum revealed by MudPIT analysis.  Proteomics 2014 Jun;14(12):1531-42. PMID:  24737727 | GPL17304 | Microarray | GSE48038 | GSM1166149 | BHI_delta_pup x BHI_WT |
| Küberl et. al. | 2014 | Küberl A, Fränzel B, Eggeling L, Polen T et al. Pupylated proteins in Corynebacterium glutamicum revealed by MudPIT analysis.  Proteomics 2014 Jun;14(12):1531-42. PMID:  24737727 | GPL17304 | Microarray | GSE48038 | GSM1166150 | BHI_WT x BHI_delta_pup |
| Kallscheuer et. al. | 2015 | Kallscheuer N, Vogt M, Kappelmann J, Krumbach K et al. Identification of the phd gene cluster responsible for phenylpropanoid utilization in Corynebacterium glutamicum. Appl Microbiol Biotechnol 2016 Feb;100(4):1871-1881. PMID:  26610800 | GPL20268 | Microarray | GSE69413 | GSM1700870 | glutamicum_control x caffeic |
| Kallscheuer et. al. | 2015 | Kallscheuer N, Vogt M, Kappelmann J, Krumbach K et al. Identification of the phd gene cluster responsible for phenylpropanoid utilization in Corynebacterium glutamicum. Appl Microbiol Biotechnol 2016 Feb;100(4):1871-1881. PMID:  26610800 | GPL20268 | Microarray | GSE69413 | GSM1700871 | glutamicum_control x caffeic |
| Kallscheuer et. al. | 2015 | Kallscheuer N, Vogt M, Kappelmann J, Krumbach K et al. Identification of the phd gene cluster responsible for phenylpropanoid utilization in Corynebacterium glutamicum. Appl Microbiol Biotechnol 2016 Feb;100(4):1871-1881. PMID:  26610800 | GPL20268 | Microarray | GSE69413 | GSM1700872 | glutamicum_control x ferulic |
| Kallscheuer et. al. | 2015 | Kallscheuer N, Vogt M, Kappelmann J, Krumbach K et al. Identification of the phd gene cluster responsible for phenylpropanoid utilization in Corynebacterium glutamicum. Appl Microbiol Biotechnol 2016 Feb;100(4):1871-1881. PMID:  26610800 | GPL20268 | Microarray | GSE69413 | GSM1700873 | glutamicum_control x ferulic |

31382874 GPL16989 Microarray GSE107921 GSM2883409 Δcg1631 x glucose Kraxner et. al. 2019

Kraxner KJ, Polen T, Baumgart M, Bott M. The conserved actinobacterial transcriptional regulator FtsR controls expression of ftsZ and further target genes and influences growth and cell division in Corynebacterium glutamicum.

BMC Microbiol 2019 Aug 5;19(1):179. PMID:

31382874 GPL16989 Microarray GSE107921 GSM2883411 glucose x Δcg1631

Kallscheuer et. al. 2015

Kallscheuer N, Vogt M, Kappelmann J, Krumbach K et al. Identification of the phd gene cluster responsible for phenylpropanoid utilization in Corynebacterium glutamicum. Appl Microbiol Biotechnol 2016 Feb;100(4):1871-1881. PMID:

| Kallscheuer et. al. | 2015 | Kallscheuer N, Vogt M, Kappelmann J, Krumbach K et al. Identification of the phd gene cluster responsible for phenylpropanoid utilization in Corynebacterium glutamicum. Appl Microbiol Biotechnol 2016 Feb;100(4):1871-1881. PMID:  26610800 | GPL20268 | Microarray | GSE69413 | GSM1700876 | glutamicum_control x p-coumaric |
| --- | --- | --- | --- | --- | --- | --- | --- |
| Kallscheuer et. al. | 2015 | Kallscheuer N, Vogt M, Kappelmann J, Krumbach K et al. Identification of the phd gene cluster responsible for phenylpropanoid utilization in Corynebacterium glutamicum. Appl Microbiol Biotechnol 2016 Feb;100(4):1871-1881. PMID:  26610800 | GPL20268 | Microarray | GSE69413 | GSM1700877 | glutamicum_control x p-coumaric |
| Kallscheuer et. al. | 2015 | Kallscheuer N, Vogt M, Kappelmann J, Krumbach K et al. Identification of the phd gene cluster responsible for phenylpropanoid utilization in Corynebacterium glutamicum. Appl Microbiol Biotechnol 2016 Feb;100(4):1871-1881. PMID:  26610800 | GPL20268 | Microarray | GSE69413 | GSM1700878 | glutamicum_control x 4-hydroxybenzoic |
| Kallscheuer et. al. | 2015 | Kallscheuer N, Vogt M, Kappelmann J, Krumbach K et al. Identification of the phd gene cluster responsible for phenylpropanoid utilization in Corynebacterium glutamicum. Appl Microbiol Biotechnol 2016 Feb;100(4):1871-1881. PMID:  26610800 | GPL20268 | Microarray | GSE69413 | GSM1700879 | glutamicum_control x 4-hydroxybenzoic |
| Kallscheuer et. al. | 2015 | Kallscheuer N, Vogt M, Kappelmann J, Krumbach K et al. Identification of the phd gene cluster responsible for phenylpropanoid utilization in Corynebacterium glutamicum. Appl Microbiol Biotechnol 2016 Feb;100(4):1871-1881. PMID:  26610800 | GPL20268 | Microarray | GSE69413 | GSM1700880 | glutamicum_control x 3-(4-hydroxyphenyl-)propion |
| Kallscheuer et. al. | 2015 | Kallscheuer N, Vogt M, Kappelmann J, Krumbach K et al. Identification of the phd gene cluster responsible for phenylpropanoid utilization in Corynebacterium glutamicum. Appl Microbiol Biotechnol 2016 Feb;100(4):1871-1881. PMID:  26610800 | GPL20268 | Microarray | GSE69413 | GSM1700881 | glutamicum_control x 3-(4-hydroxyphenyl-)propion |
| Kallscheuer et. al. | 2015 | Kallscheuer N, Vogt M, Kappelmann J, Krumbach K et al. Identification of the phd gene cluster responsible for phenylpropanoid utilization in Corynebacterium glutamicum. Appl Microbiol Biotechnol 2016 Feb;100(4):1871-1881. PMID:  26610800 | GPL20268 | Microarray | GSE69413 | GSM1700882 | glutamicum_control x cinnamic |
| Kallscheuer et. al. | 2015 | Kallscheuer N, Vogt M, Kappelmann J, Krumbach K et al. Identification of the phd gene cluster responsible for phenylpropanoid utilization in Corynebacterium glutamicum. Appl Microbiol Biotechnol 2016 Feb;100(4):1871-1881. PMID:  26610800 | GPL20268 | Microarray | GSE69413 | GSM1700883 | glutamicum_control x cinnamic |
| Kallscheuer et. al. | 2015 | Kallscheuer N, Vogt M, Kappelmann J, Krumbach K et al. Identification of the phd gene cluster responsible for phenylpropanoid utilization in Corynebacterium glutamicum. Appl Microbiol Biotechnol 2016 Feb;100(4):1871-1881. PMID:  26610800 | GPL20268 | Microarray | GSE69412 | GSM1700868 | control x pulse |
| Kallscheuer et. al. | 2015 | Kallscheuer N, Vogt M, Kappelmann J, Krumbach K et al. Identification of the phd gene cluster responsible for phenylpropanoid utilization in Corynebacterium glutamicum. Appl Microbiol Biotechnol 2016 Feb;100(4):1871-1881. PMID:  26610800 | GPL20268 | Microarray | GSE69412 | GSM1700869 | control x pulse |
| Pfeifer et. al. | 2016 | Pfeifer E, Hünnefeld M, Popa O, Polen T et al. Silencing of cryptic prophages in  Corynebacterium glutamicum. Nucleic Acids Res 2016 Dec 1;44(21):10117-10131. PMID:  27492287 | GPL20268 | Microarray | GSE80674 | GSM2133194 | wt x cgpS-N |
| Pfeifer et. al. | 2016 | Pfeifer E, Hünnefeld M, Popa O, Polen T et al. Silencing of cryptic prophages in  Corynebacterium glutamicum. Nucleic Acids Res 2016 Dec 1;44(21):10117-10131. PMID:  27492287 | GPL20268 | Microarray | GSE80674 | GSM2133193 | cgpS-N x wt |
| Pfeifer et. al. | 2016 | Pfeifer E, Hünnefeld M, Popa O, Polen T et al. Silencing of cryptic prophages in  Corynebacterium glutamicum. Nucleic Acids Res 2016 Dec 1;44(21):10117-10131. PMID:  27492287 | GPL20268 | Microarray | GSE80674 | GSM2133195 | wt x cgpS-N |

26610800 GPL20268 Microarray GSE69413 GSM1700874 glutamicum_control x Δcg0343 Kallscheuer et. al. 2015

Kallscheuer N, Vogt M, Kappelmann J, Krumbach K et al. Identification of the phd gene cluster responsible for phenylpropanoid utilization in Corynebacterium glutamicum. Appl Microbiol Biotechnol 2016 Feb;100(4):1871-1881. PMID:

26610800 GPL20268 Microarray GSE69413 GSM1700875 glutamicum_control x Δcg0343

Schulte et. al. 2016

Schulte J, Baumgart M, Bott M. Identification of the cAMP phosphodiesterase CpdA as novel key player in cAMP-dependent regulation in

Corynebacterium glutamicum. Mol Microbiol

| Schulte et. al. | 2016 | Schulte J, Baumgart M, Bott M. Identification of the cAMP phosphodiesterase CpdA as novel key player in cAMP-dependent regulation in Corynebacterium glutamicum. Mol Microbiol  2017 Feb;103(3):534-552. PMID: 27862445 | GPL20268 | Microarray | GSE81004 | GSM2140566 | ∆cpdA x pEKEx2 |
| --- | --- | --- | --- | --- | --- | --- | --- |
| Schulte et. al. | 2016 | Schulte J, Baumgart M, Bott M. Identification of the cAMP phosphodiesterase CpdA as novel key player in cAMP-dependent regulation in Corynebacterium glutamicum. Mol Microbiol  2017 Feb;103(3):534-552. PMID: 27862445 | GPL20268 | Microarray | GSE81004 | GSM2140567 | pEKEx2 x pEKEx2-cpdA |
| Schulte et. al. | 2016 | Schulte J, Baumgart M, Bott M. Identification of the cAMP phosphodiesterase CpdA as novel key player in cAMP-dependent regulation in Corynebacterium glutamicum. Mol Microbiol  2017 Feb;103(3):534-552. PMID: 27862445 | GPL20268 | Microarray | GSE81004 | GSM2140568 | pEKEx2 x pEKEx2-cpdA |
| Schulte et. al. | 2016 | Schulte J, Baumgart M, Bott M. Identification of the cAMP phosphodiesterase CpdA as novel key player in cAMP-dependent regulation in Corynebacterium glutamicum. Mol Microbiol  2017 Feb;103(3):534-552. PMID: 27862445 | GPL20268 | Microarray | GSE81004 | GSM2140569 | pEKEx2-cpdA x pEKEx2 |
| Baumgart et. al. | 2017 | Baumgart M, Unthan S, Kloß R, Radek A et al.  Corynebacterium glutamicum Chassis C1*:  Building and Testing a Novel Platform Host for Synthetic Biology and Industrial Biotechnology.  ACS Synth Biol 2018 Jan 19;7(1):132-144. PMID:  28803482 | GPL22561 | Microarray | GSE88717 | GSM2344666 | MB001 x C1 |
| Baumgart et. al. | 2017 | Baumgart M, Unthan S, Kloß R, Radek A et al.  Corynebacterium glutamicum Chassis C1*:  Building and Testing a Novel Platform Host for Synthetic Biology and Industrial Biotechnology.  ACS Synth Biol 2018 Jan 19;7(1):132-144. PMID:  28803482 | GPL22561 | Microarray | GSE88717 | GSM2344667 | C1 x MB001 |
| Baumgart et. al. | 2017 | Baumgart M, Unthan S, Kloß R, Radek A et al.  Corynebacterium glutamicum Chassis C1*:  Building and Testing a Novel Platform Host for Synthetic Biology and Industrial Biotechnology.  ACS Synth Biol 2018 Jan 19;7(1):132-144. PMID:  28803482 | GPL22561 | Microarray | GSE88717 | GSM2344668 | MB001 x C1 |
| Baumgart et. al. | 2017 | Baumgart M, Unthan S, Kloß R, Radek A et al.  Corynebacterium glutamicum Chassis C1*:  Building and Testing a Novel Platform Host for Synthetic Biology and Industrial Biotechnology.  ACS Synth Biol 2018 Jan 19;7(1):132-144. PMID:  28803482 | GPL22561 | Microarray | GSE88717 | GSM2344669 | C1 x MB001 |
| Hünnefeld et. al. | 2019 | Hünnefeld M, Persicke M, Kalinowski J, Frunzke J.  The MarR-Type Regulator MalR Is Involved in  Stress-Responsive Cell Envelope Remodeling in  <i>Corynebacterium glutamicum</i>. Front  Microbiol 2019;10:1039. PMID: 31164873 GPL22561 | | Microarray | GSE116655 | GSM3244904 | control x pEKEx2-malR |
| Hünnefeld et. al. | 2019 | Hünnefeld M, Persicke M, Kalinowski J, Frunzke J. The MarR-Type Regulator MalR Is Involved in  Stress-Responsive Cell Envelope Remodeling in  <i>Corynebacterium glutamicum</i>. Front  Microbiol 2019;10:1039. PMID: 31164873 GPL22561 | | Microarray | GSE116655 | GSM3244903 | pEKEx2-malR x control |
| Hünnefeld et. al. | 2019 | Hünnefeld M, Persicke M, Kalinowski J, Frunzke J. The MarR-Type Regulator MalR Is Involved in  Stress-Responsive Cell Envelope Remodeling in  <i>Corynebacterium glutamicum</i>. Front  Microbiol 2019;10:1039. PMID: 31164873 GPL22561 | | Microarray | GSE116655 | GSM3244905 | control x pEKEx2-malR |
| Küberl et. al. | 2020 | Küberl A, Mengus-Kaya A, Polen T, Bott M. The Iron Deficiency Response of Corynebacterium glutamicum and a Link to Thiamine Biosynthesis.  Appl Environ Microbiol 2020 May 5;86(10). PMID:  32144105 GPL22792 | | Microarray | GSE92348 | GSM2427953 | wt x 1uM_Fe |
| Küberl et. al. | 2020 | Küberl A, Mengus-Kaya A, Polen T, Bott M. The Iron Deficiency Response of Corynebacterium glutamicum and a Link to Thiamine Biosynthesis.  Appl Environ Microbiol 2020 May 5;86(10). PMID:  32144105 GPL22792 | | Microarray | GSE92348 | GSM2427954 | wt x 1uM_Fe |
| Küberl et. al. | 2020 | Küberl A, Mengus-Kaya A, Polen T, Bott M. The Iron Deficiency Response of Corynebacterium glutamicum and a Link to Thiamine Biosynthesis.  Appl Environ Microbiol 2020 May 5;86(10). PMID:  32144105 GPL22792 | | Microarray | GSE92348 | GSM2427955 | 1uM_Fe x wt |

2017 Feb;103(3):534-552. PMID: 27862445 GPL20268 Microarray GSE81004 GSM2140564 pEKEx2 x ∆cpdA Schulte et. al. 2016

Schulte J, Baumgart M, Bott M. Identification of the cAMP phosphodiesterase CpdA as novel key player in cAMP-dependent regulation in

Corynebacterium glutamicum. Mol Microbiol

2017 Feb;103(3):534-552. PMID: 27862445 GPL20268 Microarray GSE81004 GSM2140565 pEKEx2 x ∆cpdA

Küberl et. al. 2020

Küberl A, Mengus-Kaya A, Polen T, Bott M. The Iron Deficiency Response of Corynebacterium glutamicum and a Link to Thiamine Biosynthesis.

Appl Environ Microbiol 2020 May 5;86(10). PMID:

| Küberl et. al. | 2020 | Küberl A, Mengus-Kaya A, Polen T, Bott M. The Iron Deficiency Response of Corynebacterium glutamicum and a Link to Thiamine Biosynthesis.  Appl Environ Microbiol 2020 May 5;86(10). PMID:  32144105 GPL22792 | | Microarray | GSE92397 | GSM2427955 | WT_1uM_Fe x WT |
| --- | --- | --- | --- | --- | --- | --- | --- |
| Küberl et. al. | 2020 | Küberl A, Mengus-Kaya A, Polen T, Bott M. The Iron Deficiency Response of Corynebacterium glutamicum and a Link to Thiamine Biosynthesis.  Appl Environ Microbiol 2020 May 5;86(10). PMID:  32144105 GPL22792 | | Microarray | GSE92397 | GSM2428060 | WT x WT_1uM_Fe |
| Küberl et. al. | 2020 | Küberl A, Mengus-Kaya A, Polen T, Bott M. The Iron Deficiency Response of Corynebacterium glutamicum and a Link to Thiamine Biosynthesis.  Appl Environ Microbiol 2020 May 5;86(10). PMID:  32144105 GPL22792 | | Microarray | GSE92397 | GSM2428061 | WT_1uM_Fe x WT |
| Küberl et. al. | 2020 | Küberl A, Mengus-Kaya A, Polen T, Bott M. The Iron Deficiency Response of Corynebacterium glutamicum and a Link to Thiamine Biosynthesis.  Appl Environ Microbiol 2020 May 5;86(10). PMID: | | Microarray | GSE92397 | GSM2428062 | WT x WT_1uM_Fe |
|  |  | 32144105 | GPL22792 |  |  |  |  |
| Endres et. al. | 2018 | Endres S, Wingen M, Torra J, Ruiz-González R et al. An optogenetic toolbox of LOV-based photosensitizers for light-driven killing of bacteria. Sci Rep 2018 Oct 9;8(1):15021. PMID:  30301917 | GPL22792 | Microarray | GSE110168 | GSM2981051 | M49I_control x M49I_sample |
| Endres et. al. | 2018 | Endres S, Wingen M, Torra J, Ruiz-González R et al. An optogenetic toolbox of LOV-based photosensitizers for light-driven killing of bacteria. Sci Rep 2018 Oct 9;8(1):15021. PMID:  30301917 | GPL22792 | Microarray | GSE110168 | GSM2981052 | M49I_control x M49I_sample |
| Endres et. al. | 2018 | Endres S, Wingen M, Torra J, Ruiz-González R et al. An optogenetic toolbox of LOV-based photosensitizers for light-driven killing of bacteria. Sci Rep 2018 Oct 9;8(1):15021. PMID:  30301917 | GPL22792 | Microarray | GSE110168 | GSM2981053 | M49I_control x M49I_sample |
| Endres et. al. | 2018 | Endres S, Wingen M, Torra J, Ruiz-González R et al. An optogenetic toolbox of LOV-based photosensitizers for light-driven killing of bacteria. Sci Rep 2018 Oct 9;8(1):15021. PMID:  30301917 | GPL22792 | Microarray | GSE110168 | GSM2981054 | Pp2FbFP_sample x Pp2FbFP_control |
| Endres et. al. | 2018 | Endres S, Wingen M, Torra J, Ruiz-González R et al. An optogenetic toolbox of LOV-based photosensitizers for light-driven killing of bacteria. Sci Rep 2018 Oct 9;8(1):15021. PMID:  30301917 | GPL22792 | Microarray | GSE110168 | GSM2981055 | Pp2FbFP_control x Pp2FbFP_sample |
| Endres et. al. | 2018 | Endres S, Wingen M, Torra J, Ruiz-González R et al. An optogenetic toolbox of LOV-based photosensitizers for light-driven killing of bacteria. Sci Rep 2018 Oct 9;8(1):15021. PMID:  30301917 | GPL22792 | Microarray | GSE110168 | GSM2981056 | Pp2FbFP_control x Pp2FbFP_sample |
| Davoudi et. al. | 2019 | Davoudi CF, Ramp P, Baumgart M, Bott M. Identification of Surf1 as an assembly factor of the cytochrome bc<sub>1</sub>- |  | Microarray | GSE123974 | GSM3517977 | wt x [delta]cg2460 |
|  |  | aa<sub>3</sub> supercomplex of Actinobacteria.  Biochim Biophys Acta Bioenerg 2019 Oct  1;1860(10):148033. PMID: 31226315 GPL22792 | |  |  |  |  |
| Davoudi et. al. | 2019 | Davoudi CF, Ramp P, Baumgart M, Bott M. Identification of Surf1 as an assembly factor of  the cytochrome bc<sub>1</sub>aa<sub>3</sub> supercomplex of Actinobacteria.  Biochim Biophys Acta Bioenerg 2019 Oct  1;1860(10):148033. PMID: 31226315 GPL22792 | | Microarray | GSE123974 | GSM3517978 | wt x [delta]cg2460 |
| Davoudi et. al. | 2019 | Davoudi CF, Ramp P, Baumgart M, Bott M. Identification of Surf1 as an assembly factor of  the cytochrome bc<sub>1</sub>aa<sub>3</sub> supercomplex of Actinobacteria.  Biochim Biophys Acta Bioenerg 2019 Oct  1;1860(10):148033. PMID: 31226315 GPL22792 | | Microarray | GSE123974 | GSM3517979 | wt x [delta]cg2460 |
| Davoudi et. al. | 2019 | Davoudi CF, Ramp P, Baumgart M, Bott M. Identification of Surf1 as an assembly factor of  the cytochrome bc<sub>1</sub>aa<sub>3</sub> supercomplex of Actinobacteria.  Biochim Biophys Acta Bioenerg 2019 Oct  1;1860(10):148033. PMID: 31226315 GPL22792 | | Microarray | GSE123974 | GSM3517980 | [delta]cg2460 x wt |
| Küberl et. al. | 2020 | Küberl A, Mengus-Kaya A, Polen T, Bott M. The Iron Deficiency Response of Corynebacterium glutamicum and a Link to Thiamine Biosynthesis.  Appl Environ Microbiol 2020 May 5;86(10). PMID:  32144105 GPL22794 | | Microarray | GSE92397 | GSM2427953 | WT x WT_1uM_Fe |

32144105 GPL22792 Microarray GSE92397 GSM2427953 WT x WT_1uM_Fe Küberl et. al. 2020

Küberl A, Mengus-Kaya A, Polen T, Bott M. The Iron Deficiency Response of Corynebacterium glutamicum and a Link to Thiamine Biosynthesis.

Appl Environ Microbiol 2020 May 5;86(10). PMID:

32144105 GPL22792 Microarray GSE92397 GSM2427954 WT x WT_1uM_Fe

Küberl et. al. 2020

Küberl A, Mengus-Kaya A, Polen T, Bott M. The Iron Deficiency Response of Corynebacterium glutamicum and a Link to Thiamine Biosynthesis.

Appl Environ Microbiol 2020 May 5;86(10). PMID:

| Küberl et. al. | 2020 | Küberl A, Mengus-Kaya A, Polen T, Bott M. The Iron Deficiency Response of Corynebacterium glutamicum and a Link to Thiamine Biosynthesis.  Appl Environ Microbiol 2020 May 5;86(10). PMID:  32144105 GPL22794 | | Microarray | GSE92397 | GSM2428060 | WT x WT_1uM_Fe |
| --- | --- | --- | --- | --- | --- | --- | --- |
| Küberl et. al. | 2020 | Küberl A, Mengus-Kaya A, Polen T, Bott M. The Iron Deficiency Response of Corynebacterium glutamicum and a Link to Thiamine Biosynthesis.  Appl Environ Microbiol 2020 May 5;86(10). PMID:  32144105 GPL22794 | | Microarray | GSE92397 | GSM2428061 | WT_1uM_Fe x WT |
| Küberl et. al. | 2020 | Küberl A, Mengus-Kaya A, Polen T, Bott M. The Iron Deficiency Response of Corynebacterium glutamicum and a Link to Thiamine Biosynthesis.  Appl Environ Microbiol 2020 May 5;86(10). PMID: | | Microarray | GSE92397 | GSM2428062 | WT x WT_1uM_Fe |
|  |  | 32144105 | GPL22794 |  |  |  |  |
| Zhu | 2020 |  | GPL22794 | Microarray | GSE138827 | GSM4120124 | GABA+K2SO4 x GABA+(NH4)2SO4 |
| Zhu | 2020 |  | GPL22794 | Microarray | GSE138827 | GSM4120125 | GABA+(NH4)2SO4 x GABA+K2SO4 |
| Zhu | 2020 |  | GPL22794 | Microarray | GSE138827 | GSM4120126 | GABA+(NH4)2SO4 x GABA+K2SO4 |
| Zhu | 2020 |  | GPL22794 | Microarray | GSE138827 | GSM4120127 | GABA+K2SO4 x GABA+(NH4)2SO4 |
| Zhu | 2020 |  | GPL22794 | Microarray | GSE138828 | GSM4120128 | glucose x GABA |
| Zhu | 2020 |  | GPL22794 | Microarray | GSE138828 | GSM4120129 | GABA x glucose |
| Zhu | 2020 |  | GPL22794 | Microarray | GSE138828 | GSM4120130 | glucose x GABA |
| Zhu | 2020 |  | GPL22794 | Microarray | GSE138828 | GSM4120131 | GABA x glucose |
| Krüger et. al. | 2019 | Krüger A, Wiechert J, Gätgens C, Polen T et al.  Impact of CO<sub>2</sub>/HCO<sub>3</sub>  <sup>-</sup> Availability on Anaplerotic Flux in  Pyruvate Dehydrogenase Complex-Deficient  Corynebacterium glutamicum Strains. J Bacteriol  2019 Oct 15;201(20). PMID: 31358612 | GPL26911 | Microarray | GSE134218 | GSM3939045 | ΔaceE x ΔaceE_Δpyc |
| Krüger et. al. | 2019 | Krüger A, Wiechert J, Gätgens C, Polen T et al.  Impact of CO<sub>2</sub>/HCO<sub>3</sub>  <sup>-</sup> Availability on Anaplerotic Flux in  Pyruvate Dehydrogenase Complex-Deficient  Corynebacterium glutamicum Strains. J Bacteriol  2019 Oct 15;201(20). PMID: 31358612 | GPL26911 | Microarray | GSE134218 | GSM3939046 | ΔaceE x ΔaceE_Δpyc |
| Krüger et. al. | 2019 | Krüger A, Wiechert J, Gätgens C, Polen T et al.  Impact of CO<sub>2</sub>/HCO<sub>3</sub>  <sup>-</sup> Availability on Anaplerotic Flux in  Pyruvate Dehydrogenase Complex-Deficient  Corynebacterium glutamicum Strains. J Bacteriol  2019 Oct 15;201(20). PMID: 31358612 | GPL26911 | Microarray | GSE134218 | GSM3939047 | ΔaceE x ΔaceE_Δpyc |
| Keppel et. al. | 2019 | Keppel M, Hünnefeld M, Filipchyk A, Viets U et al. HrrSA orchestrates a systemic response to heme and determines prioritization of terminal  cytochrome oxidase expression. Nucleic Acids Res  2020 Jul 9;48(12):6547-6562. PMID: 32453397 GPL25416 | | NGS | GSE120924 | GSM3421586/SRR7977557 | WT_before_heme_induction |
| Keppel et. al. | 2019 | Keppel M, Hünnefeld M, Filipchyk A, Viets U et al. HrrSA orchestrates a systemic response to heme and determines prioritization of terminal  cytochrome oxidase expression. Nucleic Acids Res  2020 Jul 9;48(12):6547-6562. PMID: 32453397 GPL25416 | | NGS | GSE120924 | GSM3421586/SRR7977558 | WT_before_heme_induction |
| Keppel et. al. | 2019 | Keppel M, Hünnefeld M, Filipchyk A, Viets U et al. HrrSA orchestrates a systemic response to heme and determines prioritization of terminal  cytochrome oxidase expression. Nucleic Acids Res  2020 Jul 9;48(12):6547-6562. PMID: 32453397 GPL25416 | | NGS | GSE120924 | GSM3421587/SRR7977559 | hrrA_before_heme_induction |
| Keppel et. al. | 2019 | Keppel M, Hünnefeld M, Filipchyk A, Viets U et al. HrrSA orchestrates a systemic response to heme and determines prioritization of terminal  cytochrome oxidase expression. Nucleic Acids Res  2020 Jul 9;48(12):6547-6562. PMID: 32453397 GPL25416 | | NGS | GSE120924 | GSM3421587/SRR7977560 | hrrA_before_heme_induction |
| Keppel et. al. | 2019 | Keppel M, Hünnefeld M, Filipchyk A, Viets U et al. HrrSA orchestrates a systemic response to heme and determines prioritization of terminal  cytochrome oxidase expression. Nucleic Acids Res  2020 Jul 9;48(12):6547-6562. PMID: 32453397 GPL25416 | | NGS | GSE120924 | GSM3421588/SRR7977561 | WT_0.5h_after_heme_induction |
| Keppel et. al. | 2019 | Keppel M, Hünnefeld M, Filipchyk A, Viets U et al. HrrSA orchestrates a systemic response to heme and determines prioritization of terminal  cytochrome oxidase expression. Nucleic Acids Res  2020 Jul 9;48(12):6547-6562. PMID: 32453397 GPL25416 | | NGS | GSE120924 | GSM3421588/SRR7977562 | WT_0.5h_after_heme_induction |

32144105 GPL22794 Microarray GSE92397 GSM2427954 WT x WT_1uM_Fe Küberl et. al. 2020

Küberl A, Mengus-Kaya A, Polen T, Bott M. The Iron Deficiency Response of Corynebacterium glutamicum and a Link to Thiamine Biosynthesis.

Appl Environ Microbiol 2020 May 5;86(10). PMID:

32144105 GPL22794 Microarray GSE92397 GSM2427955 WT_1uM_Fe x WT

Keppel et. al. 2019

Keppel M, Hünnefeld M, Filipchyk A, Viets U et al. HrrSA orchestrates a systemic response to heme and determines prioritization of terminal cytochrome oxidase expression. Nucleic Acids Res

| Keppel et. al. | 2019 | Keppel M, Hünnefeld M, Filipchyk A, Viets U et al. HrrSA orchestrates a systemic response to heme and determines prioritization of terminal  cytochrome oxidase expression. Nucleic Acids Res  2020 Jul 9;48(12):6547-6562. PMID: 32453397 GPL25416 | | NGS | GSE120924 | GSM3421590/SRR7977565 | WT_4h_after_heme_induction |
| --- | --- | --- | --- | --- | --- | --- | --- |
| Keppel et. al. | 2019 | Keppel M, Hünnefeld M, Filipchyk A, Viets U et al. HrrSA orchestrates a systemic response to heme and determines prioritization of terminal  cytochrome oxidase expression. Nucleic Acids Res  2020 Jul 9;48(12):6547-6562. PMID: 32453397 GPL25416 | | NGS | GSE120924 | GSM3421590/SRR7977566 | WT_4h_after_heme_induction |
| Keppel et. al. | 2019 | Keppel M, Hünnefeld M, Filipchyk A, Viets U et al. HrrSA orchestrates a systemic response to heme and determines prioritization of terminal  cytochrome oxidase expression. Nucleic Acids Res  2020 Jul 9;48(12):6547-6562. PMID: 32453397 GPL25416 | | NGS | GSE120924 | GSM3421591/SRR7977567 | hrrA_4h_after_heme_induction |
| Keppel et. al. | 2019 | Keppel M, Hünnefeld M, Filipchyk A, Viets U et al. HrrSA orchestrates a systemic response to heme and determines prioritization of terminal cytochrome oxidase expression. Nucleic Acids Res | | NGS | GSE120924 | GSM3421591/SRR7977568 | hrrA_4h_after_heme_induction |
|  |  | 2020 Jul 9;48(12):6547-6562. PMID: 32453397 | GPL25416 |  |  |  |  |
| Pfeifer et. al. | 2019 |  | GPL25746 | NGS | GSE122249 | GSM3462608 | Pipecolic |
| Pfeifer et. al. | 2019 |  | GPL25746 | NGS | GSE122249 | GSM3462609 | Pipecolic |
| Pfeifer et. al. | 2019 |  | GPL25746 | NGS | GSE122249 | GSM3462610 | Proline |
| Pfeifer et. al. | 2019 |  | GPL25746 | NGS | GSE122249 | GSM3462611 | Proline |
| Pfeifer et. al. | 2019 |  | GPL25746 | NGS | GSE141132 | GSM4195902 | CgpS_binding |
| Pfeifer et. al. | 2019 |  | GPL25746 | NGS | GSE141132 | GSM4195903 | CgpS_binding |
| Pfeifer et. al. | 2019 |  | GPL25746 | NGS | GSE141132 | GSM4195904 | CgpS_binding |
| Lee et. al. | 2013 | Lee JY, Seo J, Kim ES, Lee HS et al. Adaptive evolution of Corynebacterium glutamicum resistant to oxidative stress and its global gene expression profiling. Biotechnol Lett 2013  May;35(5):709-17. PMID: 23288296 | GPL16168 | NGS | GSE41232 | GSM1011518 | wt |
| Lee et. al. | 2013 | Lee JY, Seo J, Kim ES, Lee HS et al. Adaptive evolution of Corynebacterium glutamicum resistant to oxidative stress and its global gene expression profiling. Biotechnol Lett 2013  May;35(5):709-17. PMID: 23288296 | GPL16168 | NGS | GSE41232 | GSM1011519 | wt |

2020 Jul 9;48(12):6547-6562. PMID: 32453397 GPL25416 NGS GSE120924 GSM3421589/SRR7977563 hrrA_0.5h_after_heme_induction Keppel et. al. 2019

Keppel M, Hünnefeld M, Filipchyk A, Viets U et al. HrrSA orchestrates a systemic response to heme and determines prioritization of terminal cytochrome oxidase expression. Nucleic Acids Res

2020 Jul 9;48(12):6547-6562. PMID: 32453397 GPL25416 NGS GSE120924 GSM3421589/SRR7977564 hrrA_0.5h_after_heme_induction

Lee et. al. 2013

Lee JY, Seo J, Kim ES, Lee HS et al. Adaptive evolution of Corynebacterium glutamicum resistant to oxidative stress and its global gene expression profiling. Biotechnol Lett 2013

May;35(5):709-17. PMID: 23288296 GPL16168 NGS GSE41232 GSM1011520 10_mM_H2O2_adapted_(HA)
